# Research progress of exosomes from different sources in myocardial ischemia

**DOI:** 10.3389/fcvm.2024.1436764

**Published:** 2024-09-12

**Authors:** Huan Yan, Huang Ding, Ruo-Xi Xie, Zhi-Qing Liu, Xiao-Qian Yang, Ling-Li Xie, Cai-Xia Liu, Xiao-Dan Liu, Li-Yuan Chen, Xiao-Ping Huang

**Affiliations:** ^1^ Hunan Provincial Key Laboratory for Prevention and Treatment of Integrated Traditional Chinese and Western Medicine on Cardio-Cerebral Diseases, Hunan University of Chinese Medicine, Changsha, China; ^2^ Changde Hospital, Xiangya School of Medicine, Central South University, Hunan, China

**Keywords:** exosomes, different sources, myocardial ischemia, effects, mechanism, diagnosis and treatment

## Abstract

Ischemic heart disease refers to the imbalance between the supply and demand of myocardial blood; it has various causes and results in a class of clinical diseases characterized by myocardial ischemia (MI). In recent years, the incidence of cardiovascular disease has become higher and higher, and the number of patients with ischemic heart disease has also increased year by year. Traditional treatment methods include drug therapy and surgical treatment, both of which have limitations. The former maybe develop risks of drug resistance and has more significant side effects, while the latter may damage blood vessels and risk infection. At this stage, a new cell-free treatment method needs to be explored. Many research results have shown that exosomes from different cell sources can protect the ischemic myocardium via intercellular action methods, such as promoting angiogenesis, inhibiting myocardial fibrosis, apoptosis and pyroptosis, and providing a new basis for the treatment of MI. In this review, we briefly introduce the formation and consequences of myocardial ischemia and the biology of exosomes, and then focus on the role and mechanism of exosomes from different sources in MI. We also discuss the role and mechanism of exosomes pretreated with Chinese and Western medicines on myocardial ischemia. We also discuss the potential of exosomes as diagnostic markers and therapeutic drug for MI.

## 1 Introduction

Cardiovascular disease has always been a disease that severely endangers the health of people around the world, and ischemic heart disease is one of the main reasons for high human mortality (1, 2). Myocardial ischemia (MI) is mainly caused by coronary artery stenosis, leading to continuous ischemia and hypoxia in the blood vessels, and finally insufficient myocardial energy and metabolic disorders, making the heart in a pathological state such that it cannot work normally (3, 4). MI can cause a series of adverse consequences such as (acute) myocardial infarction (5) and myocardial ischemia-reperfusion injury (MI/RI) (6). The treatment of an ischemic myocardium usually relies on the dual traditional therapy of angiogenesis and drug therapy, and the therapeutic effect is usually limited by myocardial reperfusion and vascular resistance (7). Therefore, the search for an effective treatment is ongoing, and many research results have confirmed that exosomes play an important role in the treatment of ischemic myocardium (8).

**Figure 1:**
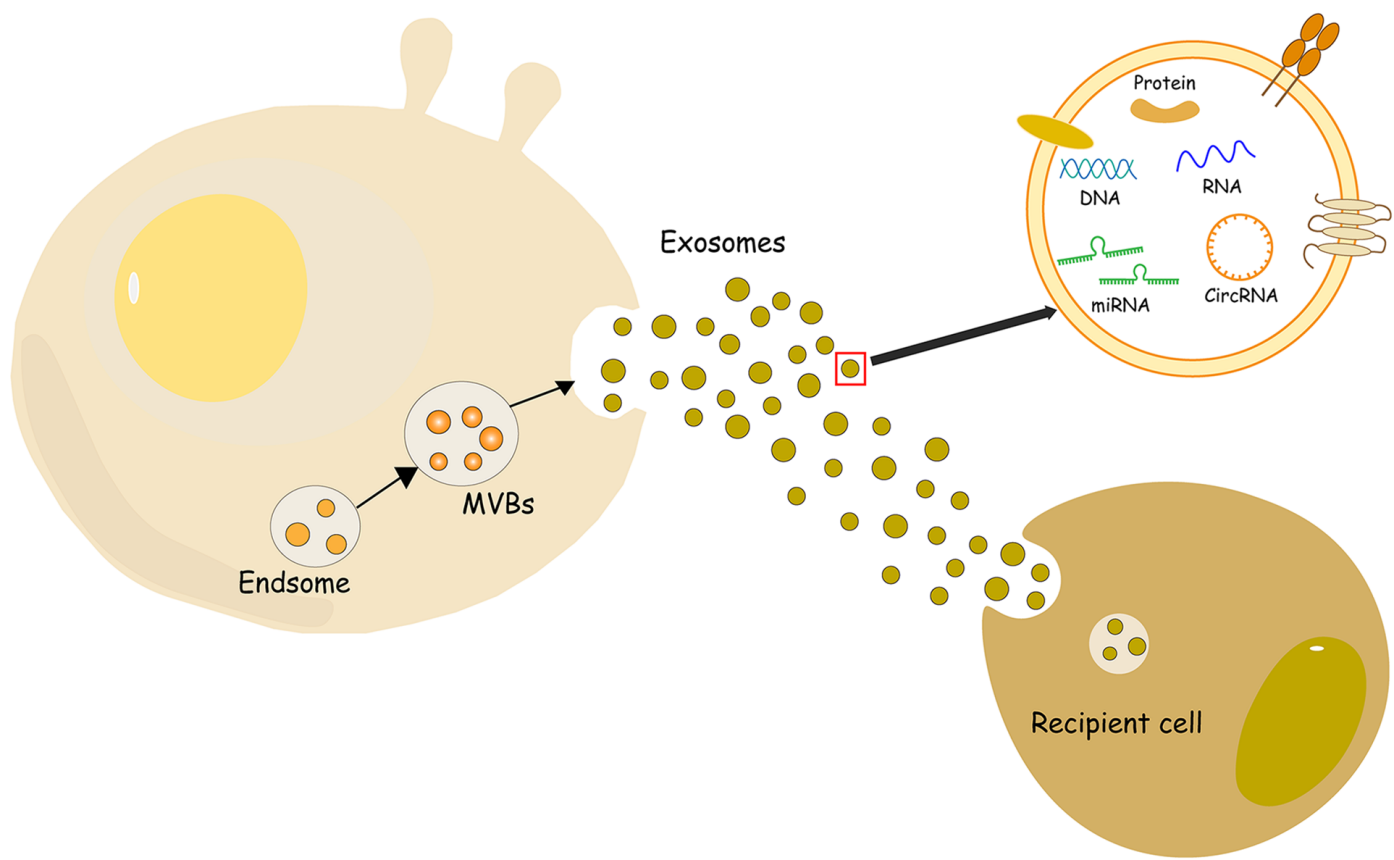
Schematic representation of the biogenesis of exosomes. MVBs, multivesicular bodies.

Extracellular vesicles (EV) are small vesicles with bilayer lipid membranes that can be divided into three subtypes: exosomes, microvesicles (MV), and apoptotic bodies (9). Exosomes are small lipid bilayer vesicles, usually 30–100 nm in size, which are formed by the intracellular budding of the endodermal vesicle membrane of multivesicular bodies (MVBs) (10) (Figure 1). MV is an EV formed by the direct outward budding or extrusion of the cell plasma membrane, with a diameter of usually 100 nm–1 μm (11). Apoptotic bodies are released as fragments of apoptosis and are thought to play a role in immunomodulation and inflammation in the tumor microenvironment. They are usually classified into larger apoptotic bodies (−1–5 μm) and smaller apoptotic vesicles (−100–1,000 nm) according to their diameter (12). Exosomes can use the endocytosis pathway to secrete lipids, protein, DNA, mRNA, and other RNA signaling biomolecules, such as microRNA (miRNA), circular RNA (circRNA), long non-coding RNA (lncRNA) and small interfering RNA (siRNA) to the recipient cells, mediating the triggering of the microenvironment of the recipient cells (13, 14). Exosomes have many advantages such as high abundance, strong biocompatibility, low immunogenicity, and strong plasticity, and they can form from a variety of different types of cells (15). Exosomes also have various biological functions, such as regulating intercellular communication (16), participating in oxidative stress (17), and resisting inflammatory responses (18). At present, the main methods for exosome separation are ultracentrifugation, ultrafiltration, gel exclusion chromatography, polymer precipitation, immunoaffinity capture, integrated microfluidic technology and other methods (19). These circulating vesicles in body fluids, including microvesicles and exosomes, have both beneficial and harmful effects on ischemia-reperfusion (IR) damage (20). In addition, the mechanism by which EVs affect immune cell metabolism during cardiac injury, as well as factors related to obesity and metabolic syndrome, which can disrupt normal EVs function, and diseases such as obesity and metabolic syndrome, which are associated with most cases of cardiovascular disease (CVD), can bias the production of EVs towards a pro-inflammatory phenotype (21). Of course, the biological origins of EVs make it particularly necessary for future studies to consider sex-related differences in addition to comorbidities associated with cardiovascular disease (22). And in the presence/absence of a cardioprotective protocol, certain cytokines stimulate the production of vesicles/exosomes. After myocardial infarction, the number of circulating extracellular vesicles carrying warning factors such as IL-1α, IL-1β and Rantes increased significantly in the inflammatory microenvironment (23). And changes in the cargo of the EV protein that can alter the release of the inflammatory factor IL-3 in the inflammatory environment, leading to a lack of protection for the heart (24). On the contrary, in the microenvironment of MI, exosomes have shown significant therapeutic application prospects by exhibiting their ability to induce angiogenesis and reduce myocardial cell apoptosis and myocardial fibrosis (25). It is precisely because of these properties that exosomes are increasingly widely used in the treatment of MI. As a carrier, exosomes and conventional vectors are often limited by tissue specificity. The targeting ability of modified exosomes has been greatly improved, so the application of modified exosomes to ischemic myocardial therapy has been considered (26). In this review, the research progress regarding the roles and mechanisms of exosomes of different cell origins in MI as well as the Intervention of drugs is summarized, providing new insights for the treatment of MI.

## 2 The effects and mechanisms of exosomes derived from heart in myocardial ischemia

Circ-HIPK3 has been shown to promote cell proliferation (27) and inhibit apoptosis (28, 29). Hypoxic cardiomyocyte released circ-HIPK3-rich exosomes to promote angiogenesis *in vivo*, and more importantly, they can also promote the proliferation of cardiac endothelial cells *in vitro*, this is based on the miR-29a/vascular endothelial growth factor A (VEGFA) signaling axis, which protects the ischemic myocardium (30). In addition, another study reported that exosomal circ-HIPK3 reduced H_2_O_2_-induced apoptosis of AC16 cardiomyocytes and promoted proliferation via the miR-33a-5p/insulin receptor substrate 1 (IRS1) axis, this provides a new insight of the pathology of acute myocardial infarction (AMI) and a potential target for the treatment of AMI (31). Overall, exosome circ-HIPK3 is beneficial to the survival and proliferation of cardiomyocytes, thereby protecting ischemic myocardium. At the same time, the expression of lncRNA AK139128 carried by myocardial cells and their exosomes after hypoxia is upregulated, and the enrichment of lncRNA AK139128 leads to the apoptosis of myocardium and proliferation of myocardial fibroblasts *in vitro* experiments. This can be reversed by downregulating the expression of lncRNA AK139128, in order to protect the ischemic myocardium (32).

According to recent studies, exosomal miRNAs play an important role in endogenous cardiomyocytes. Sun et al. (33) showed that the decreased exosomal miR-106b-3p derived from ferroptotic cardiomyocyte can activate the wingless-integration-1 (Wnt)/β-catenin pathway and cause macrophage activation to M1, thus damaging the ischemic myocardium during myocardial infarction, but the exosomes carried by cardiomyocytes treated with ferrostatin-1 (ferroptosis inhibitor) can reverse these injuries and protect the damaged myocardium. MiR-146a-5p has dual effects, causing inflammation (34) and reducing the inflammatory response (35). A recent study confirmed that miR-146a-5p has a dual effect on myocardial ischemia. Cardiomyocyte-derived exosomes miR-146a-5p can activate macrophage conversion to M1 and promote inflammation, and can also inhibit inflammation by targeting the downregulation of TNF receptor-related factor 6 (TRAF6) to protect damaged areas of the heart muscle (36). The expression of miR-19a-3p carried by cardiomyocyte exosomes is increased after myocardial infarction and H_2_O_2_ treatment, which can target hypoxia-inducible factor alpha (HIF-1α)'s downregulation to inhibit angiogenesis and endothelial cell proliferation, inhibiting miR-19a-3p can improve cardiac function and promote angiogenesis. This is a novel intercellular communication mechanism between cardiomyocytes and endothelial cells (37). Cardiomyocyte-derived exosomal miR-92a can increase the expression of α-smooth muscle actin (αSMA), αSMA increased expression is activated cardiac fibroblasts into myofibroblasts hallmark, which can protect ischemic myocardium, this experiment for the first time demonstrated that miR-92a is transferred to fibroblasts in the form of exosomes, which is crucial for the activation of cardiac fibroblasts (38). One study reported that miR-935 is a highly differentially expressed miRNA in the exosomal miRNA repertoire (exo-miR^SEL^). In the cardiac progenitor cells (CPC) surroundings, miR-935 and other exosomal miRNA members, can counteract oxidative stress-related apoptosis after MI, this is the first attempt to characterize the functional role of some exo-miR^SEL^ (39). Cardiopulmonary progenitors (CPPs) are stem cells of the heart and lung, and the protective mechanism against ischemic myocardium is unknown. Xiao et al. (40) intramuscular injection of CPPs exosomes (CPPs-Exos) promotes cardiomyocyte proliferation and angiogenesis, reduces the area of infarction and fibrosis, and improves cardiac function. To understand the underlying mechanism, they used miRNA-seq, bulk RNA-seq, and bioinformatics to find that miR-27b-3p enriched in CPPs-Exo and its target gene salt-inducible kinase 1 (Sik1) can regulate the transcriptional activity of cAMP response element-binding protein 1 (CREB1).

Cardiac fibroblast (CFs) exosomes (CFs-Exos) carrying miR-133a can effectively inhibit cardiomyocyte pyroptosis in myocardial ischemia/reperfusion (MI/R) rat models and hypoxia/reoxygenation (H/R) injury models, and suppressing ELAV-like RNA-binding protein 1 (ELAVL1) expression achieves the cardioprotective function of CFs *in vivo* and *in vitro* (41). Luo et al. (42) reported that H/R-Postcon pretreatment can up-regulate CFs exosomal miR-423-3p, target Ras-related protein Rap-2c (RAP2C), inhibit apoptosis, and exert cardioprotective effects. Sulforaphane (SFN) is an isothiocyanate that alleviate MI/RI (43). Papini et al. (44) demonstrated that continuous low-dose SFN therapy is a novel chemically-based approach for generating anti- remodeling exosomes from noncardiac fibroblasts. Exosomes derived from the pericardial adipose tissues (Pericardial-AT Exos) of transgenic mice overexpressing adipsin attenuate myocardial injury caused by myocardial infarction and relieve iron excess and lipid oxidative stress at the infarcted area (45).

In conclusion, cardiac exosomes play a protective (mainly) and damaging role in ischemic myocardial tissue (Table 1). The main myocardial protective effect is to reduce cardiomyocyte apoptosis, to promote cardiomyocyte proliferation and angiogenesis, and to resist oxidative stress; while the damage effect is mainly to promote myocardial apoptosis and inflammation. These experimental conclusions provide new targets for the diagnosis and treatment of myocardial ischemia. The discovery of vector-mediated gene editing without the need for allogeneic cells to limit immunogenicity provides a promising and novel strategy for the clinical treatment of ischemic heart injury. But in fact, the production of exosomes depends on the availability of a large number of cells, which requires cells in the heart not only to be able to release exosomes, but also not to destroy their activity or phenotype. Unfortunately, the use of autologous cardiac muscle to extract exosomes is severely limited in hospitals. Therefore, more experiments should be done in the clinic if cardiac exosomes are to be used, so as to obtain more convenient, safer, and practical cell-free therapies.

**Table 1:** Sources and components of endogenic cells-derived exosomes, their mechanisms, and their functions.

Source of exosomes	Morphology	Diameter	Exosomal maker	Component	Mechanism	Function	Experiment type	Reference
Cardiomyocytes				Circ HIPK3	miR-29a/VEGFA signaling pathway	Promote vascular regeneration and cardiac endothelial cell proliferation	*In vitro* and *in vivo*	(30)
Cardiomyocytes	Round shape with double layer membrane		CD63, HSP70	Circ-HIPK3	miR-33a-5p/IRS1 axis	Reduce cardiomyocyte apoptosis and promote cell proliferation	*In vitro*	(31)
Cardiomyocytes	Bilayer lipid membranes	100-200 nm	CD63, Hsp70, Hsp90, TSG101	LncRNA AK139128		Promote apoptosis of myocardium and proliferation of myocardial fibroblasts	*In vitro*	(32)
Ferroptotic cardiomyocyte	An elliptic sphere and central depression	50–200 nm		miR-106b-3p	Wnt/β-catenin pathway	Induce M1 polarization	*In vitro* and *in vivo*	(33)
Cardiomyocytes		30–200 nm	CD63, TSG101	miR-146a-5p	Promote M1 polarization, downregulate TRAF6	Promote inflammation, anti-inflammatory	*In vitro*	(36)
Cardiomyocytes	Cup-shaped	30–150 nm	CD63	miR-19a-3p	Downregulate HIF-1α	Inhibit endothelial cells proliferation, angiogenesis	*In vitro* and *in vivo*	(37)
Cardiomyocytes			CD81, TSG101, syntenin-1	miR-92a	Target Smad 7	Mediate post-ischemic myofibroblast activation	*In vitro* and *in vivo*	(38)
Cardiac progenitor cells		About 180 nm	CD63, CD9, HSP70, CD81	miR-935		Reduce oxidative stress and apoptosis	*In vitro*	(39)
CPPs^a^	Cup-shaped	136.9 ± 5.9 nm	CD63, Alix, Hsp70	miR-27b-3p	Target Sik1, CREB1	Promote cardiomyocyte proliferation and angiogenesis, reduce fibrosis	*In vitro* and *in vivo*	(40)
Cardiac fibroblast			CD9, CD81, TSG101	miR-133a	Suppressing ELAVL1	Inhibit cardiomyocytes pyroptosis	*In vitro* and *in vivo*	(41)
Cardiac fibroblast	Cup-shaped	About 90.25 nm	CD9, CD63, TSG101	miR-423-3p	Downregulate RAP2C	Reduce apoptosis	*In vitro* and *in vivo*	(42)
Cardiac fibroblast		100–160 nm	CD81, TSG101, Hsp70			Reduces myocardial hypertrophy, apoptosis, oxidative stress	*In vitro* and *in vivo*	(44)
Pericardial-AT^a^			CD63, TSG101	Adipsin	Target IRP2	Protect cardiomyocytes against ferroptosis, maintain iron homeostasis	*In vitro* and *in vivo*	(45)

^a^ CPPs, cardiopulmonary progenitors; Pericardial-AT, pericardial adipose tissues.

## 3 The effects and mechanisms of exosomes of mesenchymal stem cells from different sources in myocardial ischemia

Mesenchymal stem cells (MSCs) are adult stem cells with strong proliferation and differentiation capabilities that can repair cells in damaged tissues or organs (46, 47). More and more research results show that exosomes derived from MSCs have good therapeutic effects. MSC-derived exosomes (MSC-Exos) can protect the myocardial site after ischemic injury by reducing oxidative stress (48), inhibiting apoptosis and autophagy (49), and promoting angiogenesis (50, 51). They demonstrate strong reliability, safety, and efficacy in the treatment of cardiovascular disease (52). It is worth noting that external stimuli of MSC-Exos and its inclusion can alter its biological properties. Therefore, *in vitro* treatments of MSC-Exos, such as engineering modification, hypoxia/drug pretreatment, etc., can enhance the protective effect of MSC-Exos on the heart (53). However, when MI occurs, MSCs are injected into the infarcted area; although they can largely repair the myocardial injury site, they are accompanied by certain risks, such as immunogenicity and carcinogenicity (54). The following will review the role and mechanism of mesenchymal stem cell exosomes from bone marrow, adipose, umbilical cord and other sources in myocardial ischemia.

### 3.1 Exosomes derived from bone marrow mesenchymal stem cells (BMSCs-exos)

Bone marrow-derived exosomal LncRNA alleviates infarcted areas in MI. The LncRNA HCP5 (HLA complex P5) in hBMSC-Exos inhibits H/R and I/R-induced cardiomyocyte apoptosis via the miR-497/insulin-like growth factor 1 (IGF-1) axis (55). IGF1 is involved in regulating cell proliferation and apoptosis (56). BMSC-Exos lncRNA KLF3-AS1 regulates the miR-23c/signal transducer and activator of transcription 5B (STAT5B) axis, which promotes the secretion of IGF-1 by MSCs, reduces infarction size and apoptosis, thereby protecting the damaged myocardium (57). BMSC-Exos can reduce ROS production and apoptosis and induce cardiomyocyte autophagy through the AMPK/mTOR and Akt/mTOR signaling pathways, thereby reducing MIRI (58). BMSC-Exos protects myocardium by promoting moderate autophagy of cardiomyocytes and inhibiting apoptosis (59).

Exosomal miR-125b can reduce apoptosis and infarct injury caused by myocardial ischemia. BMSC-Exos containing miR-125b not only inhibits inflammation and cardiomyocyte apoptosis after MI, but also reduces the area of the myocardial infarction by targeting sirtuin 7 (SIRT7) to cardiomyocytes, thereby improving MI/RI cardiac function in rats (60). Exosomes of miR-125b-5p secreted after hypoxia preconditioning target the downregulation of the apoptotic genes p53 and BRI1-associatedreceptorkinase 1 (BAK1), significantly promoting ischemic heart repair and reducing infarction size in a mouse model of myocardial infarction (61). When BMSC-Exos is injected into infarcted myocardium, exosomal miR-125b-5p regulates the p53/B-cell lymphoma 2-interacting protein 3 (Bnip3) pathway and inhibits cardiomyocyte autophagy (62).

Other miRNAs also have important protective effects on MI, such as anti-inflammatory, anti-apoptosis, anti-oxidant stress, etc. High-mobility basal box 1 (HMGB1) can promote inflammation (63, 64), BMSC-Exos overexpression of miR-129-5p improves myocardial fibrosis, down-regulates HMGB1 expression in MI mice, reduces expression of inflammatory factors, and inhibits apoptosis (65). During myocardial I/R, BMSCs-derived exosomal miR-29c reduces excessive autophagy by inhibiting phosphatase and tensin homolog (PTEN) and activating protein kinase B (AKT)/mammalian target of rapamycin (mTOR) signaling pathway, thus protecting the heart from I/R damage (66). Toll-like receptor 4 (TLR4) plays an important role in innate immune activation of inflammatory signaling pathways (67). Hypoxia-induced BMSC-Exos miR-98-5p can down-regulate myocardial enzyme levels, inhibit oxidative stress, reduce inflammatory cell infiltration and necrotic cardiomyocyte area, inhibit TLR4, activate phosphatidylinositol-3-kinase (PI3 K)/Akt signaling pathway, and reduce the damage of inflammatory factors to ischemic myocardium tissue (68). BMSC-Exos overexpressing miR-29b-3p targets the downregulation of A Disintegrin and Metalloproteinase with Thrombospondin Motifs 16 (ADAMTS16), promotes myocardial angiogenesis and ventricular remodeling, and inhibits apoptosis in myocardial infarction rats (69). BMSC-derived exosomal miR-185 inhibits ventricular remodeling and cardiomyocyte apoptosis, and improves cardiac function in myocardial infarction mice by inhibiting SOCS2 (70). Fas ligand gene (Faslg) protects cardiomyocytes in H/R injury (71). Wnt/β-catenin signaling pathway is beneficial for ischemic myocardial repair (72). BMSC-Exos overexpression of miR-149 and let-7c inhibits Faslg expression, reduces H/R injury-induced reactive oxygen species (ROS) production, apoptosis and reduced mitochondcrial membrane potential, and protects cardiomyocytes by activating Wnt/β-catenin signaling pathway (73). *In vivo* and *in vitro* experiments have confirmed that miR-182 carried by BMSC-Exos promotes the polarization of macrophages to an anti-inflammatory phenotype through the TLR4/NF-κB/PI3 K/Akt signalling cascades, to reduce the inflammatory symptoms of myocardial tissue, enhance myocardial repair abilities, and reduce the infarced area (74). BMSC-Exos miR-143-3p reduces cardiomyocyte apoptosis and autophagy and alleviates MIRI, which is mediated by the checkpoint kinase 2 (CHK2)-Beclin2 pathway (49). It has been reported that the overexpressed exosomal miR-210 in hypoxia-injured bone marrow mesenchymal stem cells regulates PI3 K/Akt and p53 pathways by targeting apoptosis-inducible factor mitochondrial-associated 3 (AIFM3) to reduce cardiomyocyte apoptosis, thereby achieving the purpose of protecting the heart (75). BMSC-Exos miR-25-3p down-regulates the expression of pro-apoptotic protein Fas ligand (FASL) and PTEN, and miR-25-3p inhibits the expression of Enhancer of zest homologue 2 (EZH2), thereby down-regulating the expression of suppressor of cytokine signalling 3 (SOCS3), improving cardiomyocyte survival and inhibiting inflammation *in vitro* and *in vivo* (76). The overexpressed BMSC-derived exosomal miR-338 can prevent cardiomyocyte apoptosis by regulating the mitogen-activated protein kinase kinase kinase 2（MAP3K2)/c-Jun N-terminal kinase (JNK) signaling pathway and significantly repair cardiac function in a rat model of myocardial infarction (77). *In vitro* and *in vivo*, BMSC-derived exosomes of exogenous miR-132 can promote angiogenesis and further improve cardiac function by downregulating p120RasGap (RASA1) (78). These miRNAs and their downstream signaling molecules provide targets for the diagnosis and treatment of MI.

The overexpression of exosomes derived from GATA binding factor 4 (GATA-4) bone marrow mes-enchymal stem cells can not only induce the differentiation of bone marrow mesenchymal stem cells (BMSCs) into cardiomyocyte-like cells by upregulating the expression of cardiomyocyte-specific antigens, cardiac troponins T (cTnT), Connexin-43, and Desmin; they can also inhibit cardiomyocyte apoptosis, in-creasing the ejection fraction of the exosome-treated group after myocardial infarction to improve cardiac function *in vivo* (79). Fibronectin type III domain protein 5 (FNDC5) is a protein located in the cytoplasm that improves inflammatory diseases (80) and repairs infarcted myocardium (81). After activation, macrophages are usually divided into two categories. M1 macrophages are mainly involved in pro-inflammatory responses, and M2 macrophages are mainly involved in anti-inflammatory responses (82). FNDC5-BMSC-Exos can increase the expression of anti-inflammatory factors, inhibit the nuclear factor kappa-B (NF-κB) signaling pathway, and upregulate the nuclear factor erythroid 2-related factor 2 (Nrf2)/heme oxygenase-1 (HO-1) axis to regulate M2 macrophage polarization to exert anti-inflammatory effects and protect myocardial infarction (83).

Engineered bone marrow mesenchymal stem cell exosomes can enhance targeting and reduce the damage caused by MI. Macrophage migration inhibitory factor (MIF) is a highly conserved secreted protein, part of a class of pro-inflammatory cytokines that play an important role in regulating cell homeostasis and immune responses (84). Exosomes secreted by overexpressed MIF bone marrow mesenchymal stem cells (MIF-BMSC-Exos) activate adenosine 5 monophosphate-activated protein kinase (AMPK) signaling pathway, inhibit H/SD-induced mitochondrial division and cardiomyocyte apoptosis, relieve cardiac remodeling and reduce the production of reactive oxygen species, protect cardiac function (85). Zhang et al. (8) in order to improve the delivery efficiency of endothelial cells to ischemic myocardium, they used a membrane fusion method to modify endothelial cells derived from mesenchymal stem cells (MSC) with core solo cell mimics, and core solo cells mimic biologically excited MSC-EV (Mon-Exos). By mimicking the recruitment characteristics of core solo cells after MI/RI, the targeting efficiency of damaged myocardium is improved and cardiac repair is promoted.

From the above findings, we can conclude that exosomes derived from BMSCs have protective effects such as reducing the autophagy of the damaged myocardium, inhibiting myocardial cell apoptosis, reducing the occurrence of inflammation after myocardial ischemia, and reducing the area of myocardial infarction (Table 2).

**Table 2:** Sources and components of bone marrow mesenchymal stem cells-derived exosomes, their mechanisms, and their functions.

Source of exosomes	Morphology	Diameter	Exosomal maker	Component	Mechanism	Function	Experiment type	Reference
BMSCs^a^	Round- or ellipse-shaped	60–100 nm	Alix, Tsg101, CD9, CD63	LncRNA HCP5	Sponging miR-497, IGF1/PI3 K/AKT pathway	Inhibit apoptosis	*In vitro* and *in vivo*	(55)
BMSCs	Round vesicles	70–150 nm	CD63, CD81	lncRNA KLF3-AS1	miR-23c/STAT5B axis	Promote angiogenesis, protect cardiac function	*In vitro* and *in vivo*	(57)
BMSCs		50–150 nm			AMPK/mTOR and Akt/mTOR pathway	Reduce ROS and apoptosis	*In vitro* and *in vivo*	(58)
BMSCs			CD63, CD81, Alix			Promote autophagy, inhibit apoptosis	*In vitro* and *in vivo*	(59)
BMSCs	Round or oval shape	60–100 nm	CD9, CD63	miR-125b	Target SIRT7	Inhibit cardiomyocyte apoptosis, anti-inflammation and improve cardiac function	*In vivo*	(60)
BMSCs	Double-membraned structures		CD63, Alix, CD9	miR-125b-5p	p53/Bnip3 pathway	Improve autophagic flux	*In vitro* and *in vivo*	(62)
BMSCs	Round	40–150 nm	TSG101, Alix	miR-125b-5p	Target p53 and BAK1	Reduce infarct size	*In vitro* and *in vivo*	(61)
BMSCs	Round	Around 100 nm	CD9, CD63, Alix	miR-129-5p	Decrease HMGB1	Inhibit apoptosis, inflammation, improve myocardial fibrosis	*In vitro* and *in vivo*	(65)
BMSCs			CD9, CD63, Alix	miR-29c	PTEN/Akt/mTOR pathway	Inhibit autophagy	*In vitro* and *in vivo*	(66)
BMSCs			CD63, CD9	miR-98-5p	Downregulate TLR4, PI3 K/Akt pathway	Improve cardiac function, inhibit inflammation and oxidative stress	*In vivo*	(68)
BMSCs		30–150 nm	CD81, TSG101	miR-29b-3p	Downregulate ADAMTS16	Promotes angiogenesis and ventricular remodeling	*In vivo*	(69)
BMSCs	Round with complete lipid bilayer membrane	40–90 nm	CD9, CD63	miR-185	Target SOCS2	Represse ventricular remodeling	*In vivo*	(70)
BMSCs			CD63, ALIX, TSG101	miR-149, let-7c	Faslg/Wnt/β-catenin pathway	Inhibit apoptosis and ROS, increase endothelial cell proliferation and migration	*In vitro* and *in vivo*	(73)
BMSCs	a cup-shaped and double-layer membrane structure	50–150 nm	CD63, CD9, TSG101, Alix,	miR-182	TLR4/NF-κB/PI3 K/Akt signalling	Anti-inflammation, reduce infarct size	*In vitro* and *in vivo*	(74)
BMSCs	Cup-shaped	70–150 nm	CD63, CD81	miR-143-3p	CHK2-Beclin2 pathway	Reduce apoptosis and autophagy	*In vitro* and *in vivo*	(49)
BMSCs	Cup-shaped	30–150 nm.	CD63, TSG101	miR-210	AIFM3/p53 and PI3 K/Akt pathway	Inhibit cardiomyocyte apoptosis	*In vitro* and *in vivo*	(75)
BMSCs		An average diameter 100 nm	CD63, CD9, HSP70	miR-25-3p	MiR-25/EZH2/SOCS3 axis	Inhibit cardiomyocyte apoptosis, anti-inflammation	*In vitro* and *in vivo*	(76)
BMSCs	Round vesicles with intact membrane structure	30–200 nm	CD9, CD63, CD81	miR-338	MAP3K2/JNK pathway	Inhibit cardiomyocyte apoptosis, protect cardiac function	*In vitro* and *in vivo*	(77)
BMSCs	Cup-shaped	<150 nm	CD63, CD9	miR-132	Target RASA1 pathway	Promote angiogenesis	*In vitro* and *in vivo*	(78)
BMSCs				GATA-4		Inhibit cardiomyocyte apoptosis, improve cardiac function	*In vitro* and *in vivo*	(79)
BMSCs	cUp-shaped	About 100 nm	CD63, CD81, ALIX	FNDC5	Suppress NF-κB pathway, upregulate Nrf2/HO-1 axis	Anti-inflammation, promote M2 polarization	*In vitro* and *in vivo*	(83)
BMSCs		30–100 nm	CD63, CD81		AMPK pathway	Reduce apoptosis and ROS, inhibit cardiac remodeling	*In vitro* and *in vivo*	(85)
BMSCs	Round and lipid bilayer	116 nm	CD9 and Alix		Mac1/LFA1-ICAM-1 axis	Promote endothelial maturation and modulate macrophage subpopulations	*In vitro* and *in vivo*	(8)

^a^ BMSCs, bone marrow mesenchymal stem cells.

### 3.2 Adipose-derived mesenchymal stem cells exosomes (ADSCs-exos)

Exosomes secreted by adipose-derived mesenchymal stem cells (ADSCs) are a safe and effective measure to prevent inflammation and apoptosis caused by myocardial hypoxia and ischemia. Deng et al. (86) has found that the sphingosine 1-phosphate (S1P)/sphingosine kinase 1 (SK1)/sphingosine-1-phosphate receptor 1 (S1PR1) axis is involved in ADSC-Exos-mediated cardioprotection, and the upregulation of S1PR1 reduces the hypoxia-induced apoptosis of H9c2 cells, promotes macrophage M2 polarization, and inhibits transforming growth factor-beta1 (TGF-β1)-induced cardiomyocyte fibrosis, revealing that S1PR1 is one of the therapeutic targets of ADSC-Exos-mediated cardioprotection. Cui et al. (87) demonstrated that ADSC-Exos activates the Wnt/β-catenin signaling pathway, improves I/R-induced myocardial necrosis and apoptosis, and attenuates H/R-induced apoptosis; thus, it improves cardiomyocyte survival, providing a new therapeutic strategy for ischemic myocardial injury. ADSC-Exos can reduce cardiomyocyte apoptosis induced by oxidative stress (88). Exosomes enriched with Sirt6 adipose stem cells (ASCs) (S-ASC-Exos) reduce the expression levels of pyrosis-associated target proteins AIM2 and GSDMD, and increase the expression of mitosis-associated p62 and Beclin-1 to limit the progression of MIRI. In addition, S-ASC-Exos treatment significantly improves cardiac function and limits infarct size, emphasizing its cardioprotective properties. This study highlights the potential therapeutic role of Sirt6-enriched exosomes in cardioprotection (89).

Since cardiomyocytes are in the terminal differentiation stage and have little potential to divide, reducing apoptosis and hypertrophy of cardiomyocytes after injury has potential therapeutic value. Reducing the expression of the p53 upregulated modulator of apoptosis (PUMA) and ETS protooncogene 1 (ETS-1) can attenuate cardiac cell apoptosis and fibrosis (90). Lai et al. (91) reported ADSC-Exos containing miR-221/222 activates the Akt/NF-κB pathway by targeting PUMA and ETS-1, achieving the purpose of alleviating I/R-induced cardiomyocyte hypertrophy and apoptosis. Transforming growth factor-β (TGF-β) is a class of regulators of various cellular functions, including cellular immunity, and plays an important role in heart repair and remodeling (92). Mothers against decapentaplegic (Smad) proteins are a unique group of intracellular related proteins responsible for transducing signals induced by TGF-β superfamily to the nucleus. Mothers against decapentaplegic homolog (Smad) proteins have protective effects on myocardial fibrosis (93) and ventricular remodeling (94). *In vivo* experiments, ADSC-Exos was shown to target the downregulation of TGF-β receptor 2 (TGFβR2) and protect mothers against decapentaplegic homolog 2 (Smad 2) phosphorylation by delivering miR-671, enhancing the viability of OGD-treated cardiomyocytes and reducing the rate of apoptosis. *In vitro* experiments with ADSC-Exos containing miR-671-treated myocardium, a significant increase in anti-apoptotic, anti-fibrotic, and anti-inflammatory effects on the myocardium was observed (95). Studies have reported that ADSC-Exos enriched with miR-126 can inhibit the myocardial apoptosis, myocardial fibrosis, inflammation, and increase angiogenesis in the infarcted area (96). ADSC-Exos promote the proliferation and migration of microvascular endothelial cells, facilitate angiogenesis, and inhibit cardiomyocyte apoptosis. This process relies on ADSC-Exos-derived miR-205 (97). ADSC-derived exosomes loaded with Salvador's siRNA (SAV-siRNA) enter cardiomyocytes, significantly inhibiting Hippo signaling and inducing cardiomyocyte regeneration, improving post-infarction cardiac function (98).

### 3.3 Exosomes derived from umbilical cord mesenchymal stem cells (UCMSC-exos)

It has been previously reported that human umbilical cord mesenchymal stem cell (HUCMSC) exosomes exhibit cardioprotective effects in ischemic models and cardiomyocyte hypoxia injury models *in vitro*, and the mechanism has been partially investigated. H/R treatment induces severe endoplasmic reticulum stress (ERS) in H9c2 cells, which increases the expression levels of ERS markers and apoptosis markers and increases apoptosis. The anti-apoptosis effect of HUCMSC-EVs (extracellular vesicles) is partly mediated by inhibiting hyperactive ERS and activating the PI3 K/Akt signaling pathway (99). Circ-0001273 is abundant in HUCMSC-Exos and significantly downregulated after myocardial infarction in rats. The experimental results show that exosomes can improve cardiac structural disorders and cell edema by delivering circ-0001273, promote the repair and regeneration of the damaged myocardium, and inhibit myocardial cell apoptosis in ischemic environments (100). In inflammatory environments after myocardial ischemia injury, HUCMSC-Exos promotes the differentiation of pro-inflammatory fibroblasts into anti-inflammatory myofibroblasts, increases the density of myofibroblasts in the infarcted area, and reduces hypoxia *in vitro* and *in vivo* induced cardiomyocyte apoptosis (101).

Consistent with exosomes from other sources, HUCMSC exosomal miRNA is involved in ischemic myocardial protection. Song et al. (102) reported that HUCMSC-Exos-miR-23a-3p may inhibit the expression of DMT1, thereby inhibiting ferroptosis and alleviating myocardial injury. Mothers against decapentaplegic homolog 7 (Smad7) has been shown to play a key role in many important physiological activities such as cell proliferation, differentiation, and apoptosis. Wang et al. (103) had shown that HUCMSC-Exos may repair the myocardium after H/R injury by inhibiting miR-125b-5p and upregulating Smad7 expression. By downregulating sex-determining region Y-box 6 (SOX6) and inhibiting miR-19a expression, HUCMSC-Exos activates Akt and inhibits the Jun N-terminal kinase3 (JNK3)/caspase-3 axis, resulting in the reduced apoptosis of hypoxic cardiomyocytes and increased cell proliferation and migration, protecting cardiomyocytes from AMI injury (104). HUMSCs-derived exosomal miR-214-3p can promote angiogenesis in the area around infarction, reduce myocardial apoptosis, and improve cardiac function by targeting PTEN and activating the p-Akt signaling pathway to promote cardiac repair (105). NF-κB signaling pathway can mediates the expression of various pro-inflammatory factors (106). Phospholipase C beta 3 (PLCB3) is an important molecular target in promoting the activation of the nuclear transcription factor NF-κB P65 (107). HUCMSC-Exos transports miR-24-3p into macrophages, polarizes macrophages to M2 by inhibiting the activation of PLCB3/NF-κB signaling pathway, reduces post-myocardial infarction inflammation, and enhances followed reparative phase, ultimately reducing fibrosis area and improving cardiac function (108). HUCMSC-Exos delivers miR-181a to downregulate the inflammatory transcription factor c-Fos, enhances the immunomodulatory effect of miR-181a and the cell-targeting ability of HUCMSC-Exos, stimulates T cell proliferation, reduces inflammatory responses, and ultimately exerts a stronger therapeutic effect on cardiac I/R injury (109).

Engineered HUCMSC-Exos can significantly improve ischemic myocardial injury. The matrix metalloproteinases (MMPs) are a group of proteolytic enzymes (110), and the tissue matrix metalloproteinase inhibitors 2 (TIMP2) is a member of the TIMP family that regulates the proteolytic activity of MMPs and is involved in the regulation of angiogenesis, cell proliferation, and apoptosis (111). TIMP2-modified HUCMSC-Exos improves cardiac function after myocardial infarction, promotes angiogenesis, and limits extracellular matrix (ECM) remodeling by reducing collagen deposition. HUCMSC-Exos-TIMP2 also activates the Akt/Sfrp2 pathway, which inhibits apoptosis and oxidative stress processes (112). Macrophage migration inhibitor (MIF) is a pro-inflammatory cytokine (84), and exosomes of MSCs overexpressing MIF have a protective effect on the heart (113, 114). Experiments have shown that exosomes derived from migration inhibitory factor (MIF)-engineered umbilical cord mesenchymal stem cells (UCMSCs) can inhibit apoptosis, promote capillary formation and cardiomyocyte migration, and protect the heart, in part mediated by miR-133a-3p and its downstream Akt signaling pathway (115). Exosomes from HUCMSCs that overexpress stromal-derived factor 1 (SDF1) protect the ischemic myocardium by activating the PI3 K pathway, inhibiting cardiomyocyte apoptosis and autophagy, and promoting endothelial microvascular regeneration (116). IFN-γ is a cytokine that promotes immune regulation and has the functions of promoting the activation, maturation, proliferation, cytokine expression and effector of immune cells (117). Some studies have found that interferon γ exosomes (IFN-γ-Exos) generated after stimulating human umbilical cord mesenchymal stem cells with IFN-γ can promote angiogenesis and anti-apoptosis *in vitro*. IFN-γ-Exos inhibits inflammatory responses *in vivo*, promoting angiogenesis and cardiomyocyte survival, which is mediated by increasing miR-21 and downregulating BTG anti-proliferation factor 2 (BTG2) (118).

### 3.4 Other mesenchymal stem cells-derived exosomes

MSC-derived exosomes (MSC-Exos) significantly enhance the expression of miR-21-5p *in vivo* and *in vitro*, and miR-21-5p may promote the polarization of macrophages to the M2 phenotype, participate in anti-inflammatory activities and the repair of damaged myocardium (119). Excreted exosomes from MSCs pretreated with HO-1-inducers are superior to MSC-Exos in improving postinfarct cardiac function, a beneficial effect partially associated with miR-183-5p; miR-183-5p also improves cardiomyocyte senescence and inhibits SD/H-induced myocardial mitochondrial fission by regulating the high-mobility group box (HMGB)/extracellular signal-regulated kinase (ERK) signaling pathway (53). Cardiac MSC-Exos promote myocardial angiogenesis, cardiomyocyte proliferation, and capillary regeneration after myocardial infarction, and maintain cardiac function (120). Human placental mesenchymal stem cell exosomes (HPMSC-Exos) carrying miR-543 increase cardiomyocyte survival by silencing the expression of collagen type IV alpha 1 chain (COL4A1), promote the angiogenesis of cardiac microvascular endothelial cells, relieve MI, and reduce myocardial injury (3). HPMSC-Exos treated with MI model mice inhibit myocardial fibrosis, left ventricular remodeling, and inflammatory responses, modulation of gut microbiota and reduce cardiomyocyte damage (4).

In conclusion, exosomes derived from mesenchymal stem cells have a strong positive effect on ischemic myocardium. In the above research results, a variety of intracellular signaling cascades and multiple targets were found, providing potential therapeutic strategies for clinical repair of ischemic myocardium. MSC-Exos are more accessible, more abundant, and have fewer side effects than other sources of exosomes. The protective effect of MSCs on myocardial ischemia has been confirmed by a large number of experiments, and more and more studies have confirmed that the effect of MSC-Exos is similar to that of MSCs. However, mesenchymal stem cell-derived exosomes still have little understanding of the underlying mechanism of myocardial ischemia, and further research.

Based on the above experiments and studies, it can be concluded that exosomes from ADSCs, HUCMSCs, and other MSC sources mitigate post-myocardial ischemia injury and promote recovery by reducing inflammation, inhibiting apoptosis, and promoting angiogenesis (Table 3).

**Table 3:** Sources and components of ADSCs, HUMSCs and other MSCs-derived exosomes, their mechanisms, and their functions.

Source of exosomes	Morphology	Diameter	Exosomal marker	Component	Mechanism	Function	Experiment type	Reference
ADSCs^a^	Cup-shaped morphology	80–130 nm	CD63, CD81, and CD9		S1P/SK1/S1PR1 pathway, promote M2 polarization	Anti-inflammation, decrease myocardial apoptosis and fibrosis	*In vitro* and *in vivo*	(86)
ADSCs	Nanovesicles	30–100 nm	CD9, CD63, HSP70, CD81		Wnt/β-catenin pathway	Anti-apoptotic and prosurvival effects	*In vitro* and *in vivo*	(87)
ADSCs	Grape-like nanoparticle exosomes	Mean: 150 nm	CD63, CD29			Inhibit cardiomyocyte apoptosis	*In vitro*	(88)
ASCs	Discoid membrane vesicles		CD9, CD63, CD81	Sirt6	Downregulate AIM2 and GSDMD, upregulate p62 and Beclin-1	Improve cardiac function, limit infarct size	*In vitro* and *in vivo*	(89)
ADSCs	Central depression with “cup-shape”	Mean:112 nm	CD63, CD9	miR-221/222	Akt/NF-κB pathway, target PUMA and ETS-1	Inhibit cardiomyocyte hypertrophy and apoptosis	*In vitro* and *in vivo*	(91)
ADSCs	Oval shape	50–120 nm	CD63, CD81	miR-671	TGFBR2/Smad2 axis	Inhibit apoptosis, anti-fibrosis, anti-inflammation	*In vitro* and *in vivo*	(95)
ADSCs	Membrane vesicles	50–100 nm	CD9, CD63, TSG101	miR-126		Inhibit apoptosis, inflammation, fibrosis and increase angiogenesis	*In vitro* and *in vivo*	(96)
ADSCs	Cup-shaped structures	approximately 100 nm	Alix, TSG101, CD81, CD63	miR-205		Promote proliferation, facilitate angiogenesis, and inhibit apoptosis.	*In vitro* and *in vivo*	(97)
ADSCs	Cup-shaped morphology	around 108 nm	CD9, TSG101	SAV-siRNA	Hippo signaling pathway	Promotes cardiomyocyte regeneration	*In vitro*	(98)
HUCMSCs^a^	Spherical vesicles	27–139 nm	CD9, CD63		Activate PI3 K/Akt pathway	Inhibit apoptosis and ERS	*In vitro*	(99)
HUCMSCs	Round or oval with a bilayer membrane structure		CD9, CD63, CD81	Circ-0001273		Decrease apoptosis	*In vitro* and *in vivo*	(100)
HUCMSCs	Cup-shape	About 100 nm	CD9, CD63		Promote fibroblast-to-myofibroblast differentiation	Inhibit apoptosis	*In vitro* and *in vivo*	(101)
HUCMSCs	Elliptical nanovesicles	0–400 nm	CD9, CD63	miR-23a-3p	Target DMT1	Inhibit ferroptosis	*In vitro* and *in vivo*	(102)
HUCMSCs		50–270 nm	CD9, CD63, CD81	miR-125b-5p	Upregulate Smad7	Attenuate myocardial injury and apoptosis	*In vitro* and *in vivo*	(103)
HUCMSCs	Round or quasi-circular, lipid-coated tea saucer-like microvesicles	40–100 nm	CD9, CD63, Alix3	miR-19a	Akt/JNK3/caspase-3 axis, target SOX6	Increase cell proliferation, decrease apoptosis.	*In vitro* and *in vivo*	(104)
HUCMSCs	Round or disk-shaped.	30–150 nm	TSG101, CD81, CD63	miR-214-3p	Target PTEN, p-AKT signaling pathway	Promote angiogenesis, reduce myocardial apoptosis, and improve cardiac function	*In vitro* and *in vivo*	(105)
HUCMSCs	Saucer-shaped morphology	70–150 nm	CD63 and TSG101	miR-24-3p	Inhibit Plcb3/NF-κB pathway	Reduces inflammation, fibrosis	*In vitro* and *in vivo*	(108)
HUCMSCs			CD9, CD63, TSG101 and ALIX-101	miR-181a	Inhibit c-Fos	Anti-inflammation	*In vitro* and *in vivo*	(109)
HUCMSCs	The secreted membrane-bound, cup-shaped, exosome-like vesicles	40–90 nm	CD9, CD63		Akt/Sfrp2 pathway	Inhibit apoptosis and oxidative stress, improve cardiac function, promote angiogenesis	*In vitro* and *in vivo*	(112)
HUCMSCs	Cup-shaped structure	Around 100 nm	TSG101, CD81, CD63	miR-133a-3p	Akt pathway	Inhibit apoptosis and promote angiogenesis	*In vitro* and *in vivo*	(115)
HUCMSCs	Spherical structure surrounded by lipid bilayer membrane.	26–143 nm	CD63, Alix, and CD9		Upregulate SDF1, PI3 K pathway	Inhibit cardiomyocyte apoptosis and autophagy, promote angiogenesis	*In vitro* and *in vivo*	(116)
HUCMSCs	Lipid bilayer membrane encapsulated nanoparticles	30–150 nm	TSG101, CD81, CD63	miR-21	Downregulate BTG2	Promote angiogenesis, anti-apoptosis, anti-inflammation	*In vitro* and *in vivo*	(118)
Other MSCs (cell line)			CD63, CD9, TSG101, Alix	miR-21-5p	Promote M2 polarization	Anti-inflammation	*In vitro* and *in vivo*	(119)
Other MSCs (cell line)	Cup-shaped morphology with a double-layer membrane structure	50–150 nm	CD63, CD81, TGS101 and Alix	miR-183-5p	HMGB/ERK pathway	Promote angiogenesis, preserve heart function	*In vitro* and *in vivo*	(53)
Other MSCs (CMSCs)	The typical appearance of microvesicles	average size 120 nm	TSG101, CD81 and CD63			Promote myocardial angiogenesis, promote cardiomyocyte proliferation and preserve heart function	*In vitro* and *in vivo*	(120)
Other MSCs (HPMSCs^a^)	Round vesicles with complete structures	30–150 nm	TSG101, HSP70, and CD63	miR-543	Target COL4A1	Promote angiogenesis	*In vitro* and *in vivo*	(3)
Other MSCs (HPMSCs)	Complete membrane structure and low-density substances inside	60–200 nm				Anti-myocardial fibrosis, anti-inflammation, modulation of gut microbiota	*In vitro* and *in vivo*	(4)

^a^ ADSCs, adipose-derived mesenchymal stem cells; HUCMSCs, human umbilical cord mesenchymal stem cells; CMSCs, cardiac mesenchymal stem cells; HPMSCs, human placental mesenchymal stem cells.

## 4 The effects and mechanisms of circulating exosomes in myocardial ischemia

Remote ischemic preconditioning (RIPC) is known to reduce the cardiac damage caused by I/R (121), and research on whether RIPC-induced exosomes are involved in cardioprotective mechanisms is underway. However, there are conflicting results in animal and human remote regulation of vesicles. D'Ascenzo et al. (122) characterized the recovery of EVs in 30 patients randomized to (1:1) RIPC (EV-RIPC) or sham surgery (EV-naive) (NCT02195726) before and after PCI treatment. The results showed that serum EVs that were sham pretreated before percutaneous coronary intervention (PCI) reduced Erk-1/2 activation and induced STAT-3 phosphorylation to induce cardiac protection, while EVs in patients with remote ischemic preconditioning (RIPC) had no protective effect against ischemia/reperfusion injury (IRI). EV-naive and EV-RIPC did not drive cardioprotective pathways *in vitro* or *ex vivo* after PCI treatment. Specifically, PCI reduced the content of EV-naive Dusp6 mRNA, and EV treatment failed to activate typical cardioprotective pathways (123).

One study found that RIPC-induced plasma exosomes can reduce oxidative stress damage and reduce apoptosis by delivering miR-24, downregulating proapoptotic protein BIM expression; RIPC-Exos can also reduce the I/R-induced myocardial infarction area and protect the heart (124). Another study found that RIPC-induced plasma exosomal miR-126a-3p plays an anti-apoptotic role by enhancing phosphorylation of Akt and Erk1/2, activating the reperfusion injury salvage kinase (RISK) pathway, and downregulating the expression of Caspase-3 (125). RIPC-induced plasma exosomes can significantly reduce I/R-induced cardiomyocyte apoptosis, inflammatory responses, and the infarction size.

This is the cardioprotective effect mediated by HSP 90 in RIPC-induced plasma exosomes, inhibiting the complement system and JNK signaling pathway, ultimately alleviating I/R-induced myocardial injury and apoptosis by the upregulation of Bcl-2 expression and the downregulation of proapoptotic protein Bax (126). It has also been found that normal unpretreated plasma exosomes carry heat shock protein 70 (HSP 70) to protect the damaged myocardium by activating the toll-like receptor 4 (TLR4) immune pathway, improving cell survival and reducing the infarcted area (127). An experiment has shown that plasma exosomes activate NF-κB signaling to induce pro-inflammatory factor production and exacerbate AMI, it may be closely related to exosomal HSP 70 (128). PI3 K/Akt pathway can regulate apoptosis, autophagy (129) and protect the heart after IRI (130). The plasma exosomes induced by ischemic preconditioning activate the PI3 K/Akt pathway, reduce the production of inflammatory substances, reduce cell death and the myocardial infarction area, improve cardiac function, and alleviate I/R-induced damage (131).

According to recent studies, exosomal miRNAs plays an important role in plasma exosomes. MiR-939-5p has been repeatedly reported to inhibit angiogenesis, and some studies have found that knocking down miR-939-5p in plasma exosomes promotes the expression and activity of inducible nitric oxide synthase (iNOS) and nitric oxide (NO) in vascular endothelial cells, contributing to angiogenesis (132). Wang et al. (133) have shown that plasma exosomal miR-342-3p alleviates myocardial damage; this is attributed to miR-342-3p targeting SOX6 and the transcription factor EB (TFEB), exerting myocardial protection against apoptosis and autophagy. CD44 is a transmembrane glycoprotein on the cell surface of hyaluronic acid and other extracellular matrix molecules, cytokines, and growth factors, and is involved in various biological processes that depend on epigenetic plasticity, such as development, inflammation, and immune response (134). As an important factor regulating membrane formation, CD44 can enhance the expression of the plasma exosomal miR-125b-5p and miR-223-3p, mediate the uptake of caveolin 1 (CAV1), and promote angiogenesis through the fibroblast growth factor receptor 2 (FGFR2) signaling pathway (135). Rho-associated coiled-coil-containing kinase (ROCK) is a serine/threonine kinase downstream of the Rho family of GTPases, which regulates the signaling pathway of cardiovascular profibrosis (136). In addition, one study found that miR-152-5p in exosomes produced in the plasma of AMI patients targets Rho GTPase-activating protein 6 (ARHGAP6) through the ROCK signaling pathway, inhibiting cardiomyocyte apoptosis and fibrosis, reducing the inflammatory response, thereby limiting the development of AMI (137).

Circulating exosome-targeted activin A receptor type I (ACVR1) delivers miR-193a-5p to protect endothelial cells from oxidative stress (138). Insulin-like growth factor 1 receptors (IGF-1R)/nitric oxide (NO) signaling pathway can promote serum angiogenesis in patients with myocardial infarction. After co-culturing serum exosomes from patients with myocardial infarction with human umbilical vein endothelial cells, cell proliferation, migration, and angiogenesis were observed, which occurred due to the reduction in miR-143 expression, the targeting of IGF-1R, and enhanced NO production (139). It has been reported that in mouse hindlimb ischemia and AMI models, the miR-126-3p of exosomes in peripheral serum can promote microangiogenesis by targeting tuberous sclerosis complex 1 (TSC1) and regulating the rapamycin (mTOR) complex 1 (mTORC1)/HIF-1α axis, and the number of blood vessels increased significantly (140). Serum exosomes can also mediate the microangiogenesis of endothelial progenitor cells through the miR-190a-3p/C-X-C motif chemokine ligand-12 (CXCL12)/C-X-C motif chemokine receptor-4 (CXCR4) signaling pathway (141). MiR-155-5p in serum-derived exosomes promotes I/R injury, and overexpressed miR-155-5p targets neuronal-precursor-cell-expressed developmentally downregulated 4 (NEDD4) to inhibit CypD ubiquitination and exacerbate MI/RI (142).

Regarding the exosomal LINC00174 that is derived from vascular endothelial cells, *in vivo* and *in vitro* experiments have confirmed that it can target serine and arginine-rich splicing factor1 (SRSF1) expression to inhibit the expression of p53, which thus repressed the activation of AMPK and Akt and the downstream autophagy; it can also improve impaired cardiac function, myocardial vacuolation, and cardiomyocyte apoptosis caused by I/R injury (143). Some studies have found that, after myocardial infarction leads to the ischemic injury of myocardial tissue, human umbilical vein endothelial cells-derived exosomes regulate the expression of apoptosis-related proteins by activating the PI3 K/Akt signaling pathway, upregulating B-cell lymphoma 2 (Bcl-2) levels, inhibiting Bcl-2-associated X protein (Bax) expression, or inactivating caspase, thereby inhibiting apoptosis and exerting cardiac protective effects (144). MiR-27b-3p in the microvascular-endothelial-derived exosomes of the hypoxic myocardium can protect the ischemic myocardium by inhibiting the forkhead box O1 (Foxo1)/Gasdermin-D (GSDMD) signaling axis, reducing inflammatory-factor-mediated oxidative stress and pyroptosis in a H/R cell injury model and a rat I/R injury model (145). The exosomes of IL-10-deficient lose their functions of repairing the myocardium and promoting angiogenesis and cell activity, resulting in cardiomyocyte apoptosis and anti-angiogenesis. A large number of integrin-linked kinase (ILK) and ILK-mediated receptor cells activate the NF-κB pathway in the exosomes of IL-10-deficient EPCs, thereby altering the function of EPCs-derived exosomes and aggravating the inflammatory response, but knocking down the expression of EPCs-derived exosomes of ILK can reverse the inflammatory response (146).

Circulating exosomes have a cardioprotective effect. The extraction of circulating exosomes mostly uses clinical specimens. Therefore, these studies still have certain limitations: (a) The sample size is relatively small, and the results of the study may not be able to elucidate all functional miRNAs and related signaling pathways. (b) Due to the complexity of clinical features, the exclusion and inclusion criteria used in this study may also not cover all clinical situations. Therefore, larger sample sizes and more detailed inclusion criteria should be considered in future studies. (c) Residual proteins in purified exosomes may also play a role in exosome regulation. Finally, exosomes from circulating sources play a protective (primary) or damaging role in ischemic myocardial tissue. The protective effect is to inhibit cell apoptosis, reduce myocardial infarction area and inflammatory response, inhibit autophagy and pyroptosis as well as promote cell proliferation and angiogenesis, while the damaging effect is to promote inflammation and ferroptosis, exacerbate myocardial injury (Table 4).

**Table 4:** Sources and components of circulating-derived exosomes, their mechanisms, and their functions.

Source of exosomes	Morphology	Diameter	Exosomal maker	Component	Mechanism	Function	Experiment type	Reference
Circulating (Serum)			CD9, CD63, CD81		Reduced the EV-naive Dusp6 mRNA	PCI reprograms EV cargo, impairing, in EV-naive, their cardio-protective properties.	*In vitro* and *ex vitro*	(122)
Circulating (Serum)	Nano-size range vesicles		CD63, CD29, CD81		Activate STAT-3	Protect IRI	*In vitro* and *ex vitro*	(123)
Circulating (plasma)	Cup-shaped membrane-bound vesicles	50–200 nm	CD63, CD81, CD9	miR-24	Downregulate BIM	Alleviate oxidative stress and reduce apoptosis	*In vitro* and *in vivo*	(124)
Circulating (plasma)	Circular particles	50–90 nm	CD81, CD9	miR-126a-3p	RISK pathway, target Caspase-3	Inhibit apoptosis	*In vitro* and *in vivo*	(125)
Circulating (plasma)	“Cup-shaped” membrane-bound vesicles	50–200 nm	CD63, TSG101	HSP 90	Inhibit the complement system, JNK signaling pathway	Inhibit apoptosis, anti-inflammatory	*In vitro* and *in vivo*	(126)
Circulating (plasma)	Cup-shaped	<100 nm	CD63, CD81, HSP 70	HSP70	TLR4/ERK1/2/P38MAPK/HSP pathway	Improve cell survival and reduce infarct size	*In vitro* and *in vivo*	(127)
Circulating (plasma)	Round-shaped	30–100 nm	CD9, CD63, CD81		NF-κB signaling pathway	Pro-inflammatory	*In vitro*	(128)
Circulating (Serum)			CD63, CD81, CD9		PI3 K/Akt pathway	Anti-inflammation, reduce apoptosis and myocardial infarct size	*In vitro* and *in vivo*	(131)
Circulating (plasma)	Round, cup-shaped	30–100 nm	CD9, CD63, Flotillin	miR-939-5p	Target iNOS, NO	Promotes cell proliferation, migration and angiogenesis	*In vitro* and *in vivo*	(132)
Circulating (plasma)	Cup- or round-shaped	Mean 100 nm	Alix, CD63	miR-342-3p	Target SOX6 and TFEB	Anti-apoptosis, inhibit autophagy	*In vitro* and *in vivo*	(133)
Circulating (plasma)		WTSham:137.5 nm, KOSham:130.6 nm, WTMI:114.2 nm, KOMI: 105.5 nm	Alix, CD63, CD81	CD44, miR-125b-5p, miR-223-3p	FGFR2 pathway	Promote angiogenesis	*In vitro* and *in vivo*	(135)
Circulating (plasma)				miR-152-5p	ROCK signaling pathway, target ARHGAP6	Inhibits cardiomyocyte apoptosis and fibrosis, anti-inflammatory	*In vitro*	(137)
Circulating (Blood)	Bubble- like balloon shape	50–150 nm	HSP70, Alix	miR-193a-5p	Target ACVR1	Inhibit oxidative stress	*In vitro* and *in vivo*	(138)
Circulating (Serum)	Spherical shape	30–100 nm	CD9, CD63, Flotillin	miR-143	IGF-IR/NO pathway	Promote cell proliferation, migration and angiogenesis	*In vitro* and *in vivo*	(139)
Circulating (Serum)	Round, cup-shaped morphology	30–150 nm	Alix, CD63, TSG101	miR-126-3p	Target TSC1 and mTORC1/HIF-1α axis	Promote angiogenesis	*In vitro* and *in vivo*	(140)
Circulating (Serum)		around 100 nm	Alix, CD63, GST101	miR-190a-3p	miR-190a-3p/CXCR4/CXCL12 pathway	Promote angiogenesis	*In vitro* and *in vivo*	(141)
Circulating (Serum)	membranous structures	Approximately 100 nm	CD9, CD63, and ALIX	miR-155-5p	Inhibit CypD ubiquitination via targeting NEDD4.	Promote MI/RI	*In vitro* and *in vivo*	(142)

## 5 The effects and mechanisms of exosomes derived from endothelial cells in myocardial ischemia

Endothelial cells-derived exosomes have protective effects on ischemic myocardium and promote angiogenesis (147). Yu et al. (148) demonstrated with a mouse ischemia/reperfusion (I/R) model that exosome injection through the tail vein significantly reduced the extent of I/R-induced cardiac injury and prevented cardiomyocyte apoptosis. In addition, Sphingosylphosphorylcholine (SPC) was identified here as the primary mediator of the observed protective effect of vascular endothelial cells derived-exosomes (VEC-Exos). In addition, *in vitro* experiments showed that SPC mitigated myocardial I/R injury by activating the Parkin and nuclear receptor subfamily group A member 2/optneurin (NR4A2/OPTN) pathway, which in turn led to increased levels of mitochondrial autophagy within cardiomyopathy, indicating that enriched SPC exosomes promoted cardiomyocyte survival and alleviated I/R-induced cardiomyocyte apoptosis. Regarding the exosomal LINC00174 that is derived from vascular endothelial cells, *in vivo* and *in vitro* experiments have confirmed that it can target serine and arginine-rich splicing factor1 (SRSF1) expression to inhibit the expression of p53, which thus repressed the activation of AMPK and Akt and the downstream autophagy; it can also improve impaired cardiac function, myocardial vacuolation, and cardiomyocyte apoptosis caused by I/R injury (143). Some studies have found that, after myocardial infarction leads to the ischemic injury of myocardial tissue, human umbilical vein endothelial cells-derived exosomes regulate the expression of apoptosis-related proteins by activating the PI3 K/Akt signaling pathway, upregulating B-cell lymphoma 2 (Bcl-2) levels, inhibiting Bcl-2-associated X protein (Bax) expression, or inactivating caspase, thereby inhibiting apoptosis and exerting cardiac protective effects (144). MiR-27b-3p in the microvascular-endothelial-derived exosomes of the hypoxic myocardium can protect the ischemic myocardium by inhibiting the forkhead box O1 (Foxo1)/Gasdermin-D (GSDMD) signaling axis, reducing inflammatory-factor-mediated oxidative stress and pyroptosis in a H/R cell injury model and a rat I/R injury model (145). The exosomes of IL-10-deficient lose their functions of repairing the myocardium and promoting angiogenesis and cell activity, resulting in cardiomyocyte apoptosis and anti-angiogenesis. A large number of integrin-linked kinase (ILK) and ILK-mediated receptor cells activate the NF-κB pathway in the exosomes of IL-10-deficient EPCs, thereby altering the function of EPCs-derived exosomes and aggravating the inflammatory response, but knocking down the expression of EPCs-derived exosomes of ILK can reverse the inflammatory response (146). Exosomes derived from endothelial cells also have a very positive protective effect on ischemic myocardium (Table 5).

**Table 5:** Sources and components of endothelial cells-derived exosomes, their mechanisms, and their functions.

Source of exosomes	Morphology	Diameter	Exosomal maker	Component	Mechanism	Function	Experiment type	Reference
Vascular endothelial cell	Disc shape	The average size: 151.4 nm	CD63, TSG101	SPC	NR4A2/OPTN pathways	Diminish I/R-induced cardiac damage and prevented apoptosis of cardiomyocytes	*In vitro* and *in vivo*	(148)
Vascular endothelial cells	Exosome-like vesicles	60–90 nm	CD9, CD63, CD81	LINC00174	Srsf1-p53-myocardin pathway, Akt/AMPK pathway	Inhibit autophagy, cardiomyocyte vacuolation and apoptosis, improve cardiac function	*In vitro* and *in vivo*	(143)
Human umbilical vein endothelial cells	Round or oval membranous vesicles resembling a saucer or cup	100–150 nm	TSG101, CD63, CD81		PI3 K/Akt pathway	Inhibit cardiomyocyte apoptosis	*In vitro* and *in vivo*	(144)
Microvascular endothelial cells		50–150 nm	CD63, CD81, TSG101	miR-27b-3p	Foxo1/GSDMD pathway	Reduce oxidative stress and pyroptosis	*In vitro* and *in vivo*	(145)
Endothelial progenitor cells			flotillin-1, CD63		NF-κB pathway	Anti-inflammation, improve cardiac function	*In vitro* and *in vivo*	(146)

## 6 The effects and mechanisms of exosomes derived from immune cells in myocardial ischemia

Macrophages are classically activated (M1 macrophages) and alternatively activated (M2 macrophages) (82). M1 macrophages mainly play a pro-inflammatory role and destroy tissues; M2 macrophages play an anti-inflammatory role and repair damaged tissues (149). In the microenvironment of myocardial infarction, M1 macrophages can inhibit the ability of endothelial cells to generate blood vessels, aggravate the degree of myocardial infarction injury, and hinder cardiac repair, all of which are attributed to the pro-inflammatory M1-Exos released by M1 macrophages and the miR-155 they contain which downregulate the RAC1-PAK1/2 and Sirt1/AMPKα-eNOS pathways (150). Dong et al. (151) shown that inhibiting the expression of miR-21-5p in the exosomes of M1 macrophages can improve myocardial structural damage and myocardial fibrosis, relieve cardiomyocyte apoptosis, and thus achieve a protective effect on ischemic myocardium. It is also found that the downregulation of miR-21-5p inhibits ventricular remodeling after inhibiting the expression of tissue inhibitors of metalloproteinase 3 (TIMP3).

Thioredoxin-interacting protein (TXNIP) is a member of the inhibitory protein alpha family and a central regulator of glucose and lipid metabolism, involved in diabetes-related vascular endothelial dysfunction and inflammation (152). TXNIP can activate the endoplasmic reticulum, and stress-mediated nucleotide-binding oligomeric domain (NOD)-like receptor protein-3 (NLRP3) inflammatory body complex formation can lead to mitochondrial stress-induced apoptosis (153). Dai et al. (154) has found that M2-Exos carrying miR-148a mitigated inflammation by regulating downregulated TXNIP expression and deactivating the TLR4/NF-κB/NLRP3 inflammasome signaling pathway, which may provide a new basis for the treatment of MI/R injury. Son of sevenless homolog 1 (SOS1) is a key protein in intracellular signal transduction, a ubiquitous adaptor protein that plays an important role in many signal transduction pathways (155). M2-Exos can attenuate AMI-induced myocardial injury and inhibit cardiomyocyte apoptosis by downregulating the expression of SOX6 through exosomal miR-1271-5p (156). Guo et al. (157) first proposed that M2-Exos delivers miR-132-3p to endothelial cells (ECs) and enhances the angiogenesis of ECs by downregulating THBS1, thereby promoting angiogenesis after myocardial infarction. This may be a potential new approach to cardiac repair. Macrophage exosomal miR-155 mimetics target the downregulation of Sos1 expression, inhibit fibroblast proliferation, and exacerbate inflammatory responses in fibroblasts. The deletion of miR-155 ameliorates damage from cardiac rupture, thereby improving cardiac function in myocardial infarction (158).

It is well known that CD4^+^ T cell activation is beneficial for the recovery of myocardial wounds after myocardial infarction. Liu et al. (159) found that CD4^+^ T cells take up more MI dendritic cells-exosomes (DEXs) and activate and recruit a large number of CD4^+^ T cells to the myocardium's damaged area under the stimulation of MI-DEXs, which are used to improve the cardiac function of mice. In order to improve the short retention time of DEXs in the treatment of myocardial infarction, Zhang et al. (160) combined DEXs into a newly developed alginate hydrogel (Gel) delivery system, finally confirming that dendritic cells’ exosomes with alginate hydrogel (DEXs-Gel) had a better effect than a single gel or the injection of DEXs, regulating the transition of macrophages to M2 cells, resisting apoptosis, promoting angiogenesis, and increasing the thickness of the infarct wall. Regarding the therapeutic effect of cardiac function after infarction, in myocardial infarction models, the results showed that DEXs can promote angiogenesis; it was further found that this is achieved through the transmission of miR-494-3p, which is highly expressed in DEXs, but the target of miR-494-3p, the downstream signal, and the mechanism whereby angiogenesis is promoted have not been identified (161). Regulatory-T-cell-derived exosomes (Tregs-Exos) reduce apoptosis and infarct size by mediating macrophage polarization to M2 (162). The activated CD4^+^ T-cell-derived exosomal miR-142-3p regulates the adenomatous polyposis coli (APC)/glycogen synthase kinase-β (GSK-β)-β-catenin signaling cascade to activate WNT signaling pathway and fibroblasts, play a pro-fibrotic role, and lead to rational ventricular remodeling in heart disease. Therefore, miR-142-3p targeting the downregulation of exosomes derived from CD4^+^ T cell activation may be a promising basis for the treatment of adverse cardiac remodeling after myocardial infarction (163).

In immune cells, exosomes derived from M1 macrophages are negatively regulated for ischemic myocardial repair. The study of its mechanism can provide new insights into the functional significance of M1 macrophages and their derived exosomes in angiogenesis and cardiac repair, and also provide potential targets for regulating negative regulation. Exosomes derived from M2 macrophages and T cells can protect and repair ischemic myocardium. The gel binding of exosomes enhances the retention of DEXs *in vivo* after myocardial infarction, improves its therapeutic effect, and also provides experimental reference for other researchers. Finally, we can see that exosomes derived from immune cells have both protective functions and injury effects on ischemic myocardium. The protective function mainly involves reducing inflammation, apoptosis and Ca2^+^ overload, promoting vascular regeneration; the injury effect attributes to pro-inflammatory, ventricular remodeling, cardiomyocyte apoptosis the aggravation of cardiac dysfunction (Table 6).

**Table 6:** Sources and components of immune cells-derived exosomes, their mechanisms, and their functions.

Source of exosomes	Morphology	Diameter	Exosomal maker	Component	Mechanism	Function	Experiment type	Reference
M1 macrophages	Cup-shaped membrane-bound vesicles	30–150 nm	Alix, CD63, CD81	miR-155	Downregulate the RAC1–PAK1/2 and Sirt1/AMPKα-eNOS pathways	Inhibit angiogenesis and cardiac repair, exacerbate cardiac dysfunction	*In vitro* and *in vivo*	(150)
M1 Macrophages	Saucer-like or hemispherical with concave	40–100 nm	CD63, CD81	miR-21-5p	Target TIMP3	Promote ventricular remodeling, myocardial fibrosis and cardiomyocyte apoptosis	*In vitro* and *in vivo*	(151)
M2 Macrophages	Circle shape	100 nm	CD63, CD81, TSG101	miR-148a	Down-regulate TXNIP, inactivate TLR4/NF-κB/NLRP3 pathway	anti-inflammation	*In vitro* and *in vivo*	(154)
M2 Macrophages	Round particles with a double-layer membrane.	80–100 nm		miR-1271-5p	Downregulate SOX6	Anti-apoptosis	*In vitro* and *in vivo*	(156)
M2 Macrophages	Double-concave disks	100 nm	Alix, TSG101, CD63	miR-132-3p		Promote angiogenesis	*In vitro* and *in vivo*	(157)
Macrophages		40–100 nm	CD68, Alix	miR-155	Downregulate Sos1	Pro-inflammatory	*In vitro* and *in vivo*	(158)
Dendritic cells		30–100 nm	CD63, Alix			Improve cardiac function	*In vitro* and *in vivo*	(159)
Dendritic cells		106.5 nm	CD63, Alix		Promote M2 polarization	Improve cardiac function	*In vitro* and *in vivo*	(160)
Dendritic cells				miR-494-3p		Promote angiogenesis	*In vitro* and *in vivo*	(161)
Regulatory T cells			TSG101, CD63		Promote M2 polarization	Reduce apoptosis and infarct size	*In vitro* and *in vivo*	(162)
CD4^+^ T cells	Round or “cup-shaped” delineated by a lipid bilayer	30–150 nm	CD81, CD63, TSG101	miR-142-3p	APC-GSK-β-β-catenin pathway	Promote post-ischemic cardiac fibrosis	*In vitro* and *in vivo*	(163)

## 7 The effects and mechanisms of exosomes derived from induced cells in myocardial ischemia

Exosomes derived from induced pluripotent stem cells (iPS-Exos) can reduce the apoptosis of MI/R rat cardiomyocytes and H9c2 cells, and the protective effect of iPS-Exos on H9c2 cells from apoptosis is mediated by the secretion of protective miRNAs (miR-21 and miR-210) (164). Exosomes from iPS-derived cardiomyocytes (iCM-Exos) inhibit apoptosis and myocardial fibrosis in mouse MI models, enhance autophagy, and protect survival of hypoxic cardiomyocytes (165). Human-induced pluripotent stem cell-derived exosomes (hiPSCs-Exos) significantly inhibited hypoxic injury-mediated apoptosis and poor ventricular remodeling in porcine infarction models, promoted cardiac angiogenesis, and protected cardiac function (166). Exosomes secreted by iCMs overexpressing cyclin D2 can promote cardiomyocyte proliferation (167). HiPSC-derived endothelial cell (hiPSC-ECs) exosomes enriched with miR-100-5p can mediate the protein phosphatase 1β (PP-1β)/Phospholamban (PLB) axis to protect a damaged myocardium, maintain intracellular Ca2^+^ homeostasis to reduce apoptosis, promote angiogenesis, and limit left ventricular remodeling (168).

## 8 The intervention effects of drugs on myocardial ischemia by regulating exosomes

Astragaloside IV (AS-IV)-mediated MSC-Exos can significantly increase the angiogenesis ability of cardiomyocytes after OGD/R, enhance cell migration ability, and prevent OGD/R-induced apoptosis, and reduce the inflammatory response and myocardial injury, weaken the secondary fiber proliferation response, significantly reduce myocardial hypertrophy, and significantly reduce collagen deposition, thus alleviating myocardial injury and fibrosis in AMI rats (169). Coincidentally, another study confirmed that astragaloside IV-induced BMSCs exosomes (AS-IV-BMSC-Exos) can reduce the tissue disorder of myocardial infarction area and myocardial tissue collagen deposition, increase the number of tubes formed, and protect the cardiac function of myocardial infarction mice through the miR-411/HIF-1a axis (170). Exosomes derived from human ADSCs combined with astragalus polysaccharide can significantly promote angiogenesis, reduce cardiomyocyte apoptosis and improve its survival rate. Its myocardial protection may be achieved by activating PI3 K/Akt pathway (171). The pretreated Tongxinluo-pretreated mesenchymal stem cells derived exosomes (MSCs_TXL_-Exos) group had better therapeutic effects in anti-apoptosis, anti-inflammation, improving LVEF, and reducing infarct area. Further exosomal miRNAs analysis showed that miR-146a-5p in MSCs_TXL_-Exos mediated cardioprotective effect by inhibiting Interleukin 1 Receptor Associated Kinase 1 (IRAK1)/NF-κB signaling pathway (172). After AMI injury for 30 min, exosomes derived from BMSCs were first injected into the heart around the infarction, followed by the injection of hypoxic combined with Tongxinluo pretreated BMSCs through the tail vein on the 3rd day after AMI, the combination of exosomes and pretreated MSCs in an orderly transplantation can effectively promote cardiac repair after myocardial infarction, such as significantly increasing LVEF and LVFS, significantly improving AMI-induced left ventricular dilation, reducing infarct area and collagen area, and showing the highest arterial and capillary density (173). The main components of Suxiao Jiuxin pill (SJP) include tetramethylpyrazine (TMP) and borneol (BOR), the treatment of Chinese patent medicine SJP shows higher stimulation of cardiac MSC-derived exosome secretion in Cardiac mesenchymal stem cells (CMSCs) than single-drug TMP or BOR, thereby accelerating cardiomyocyte reproduction, promoting cardiac repair, and having stronger cardiac protection ability (174). Another study also demonstrated that exosomes treated with quick-acting heart-saving pills (SJP) increased trimethylation of histone 3 lysine 27 in HL-1 cells (H3K27me3), which promoted myocardial proliferation (175).

Cardiomyocytes pretreated with epigallocatechin gallate (EGCG) can up-regulate exosomal miR-30a to increase cardiomyocytes viability, inhibit apoptosis and autophagy-related proteins, including Bcl-2 and Beclin-1, and can significantly reduce myocardial injury after AMI (176). Ginsenoside Rh2 (Rh2) pretreatment of BMSCs-derived exosomes can enhance the homing of exosomes to cardiomyocytes, reduce the nuclear translocation and activation of NF-κB p65 and NLRP3 inflammatory bodies, thereby improving the inflammatory microenvironment of OGD (177). It is worth noting that the exosomes secreted by pretreated with irisin BMSCs (Irisin-BMSCs-Exos) can significantly improve cell viability of cardiomyocytes caused by H/R injury, and reduce pyroptosis, oxidative stress and and inflammatory cytokines IL-1β and IL-18 by suppressing NLRP3 (178). Danhong Injection-induced endothelial exosomes (DHI-exo) into the myocardial infarction area of MI Mouse can inhibit the apoptosis proteins (p53, Bak, Bax) of cardiomyocytes, thereby protecting the damaged myocardium, which is mediated by increasing the expression levels of miR-125b and regulating p53 signaling pathway (179). Ginsenoside Re was found to significantly reduce cardiac injury caused by iron sagging during myocardial I/R injury. To determine how ginsenoside Re regulates iron apoptosis, the researchers isolated exosomes from VEGFR2 + endothelial progenitor cells after I/R injury. By luciferase reporting and qRT-PCR, miR-144-3p was found to be upregulated in myocardial I/R injury, its downstream was the solute carrier family 7 member 11 (SLC7A11). Ginsenoside Re reduced myocardial I/R-induced iron sagging by miR-144-3p/SLC7A11 (180). MiR-223-5p in MSC-Exos pretreated with tanshinone IIA inhibits C-C chemokine receptor 2 (CCR2) activation, thereby reducing core solo cell infiltration, promoting angiogenesis, and alleviating myocardial I/R injury (181). MSCs-Exos pretreated with oridonin protects cardiomyocytes from ischemia-reperfusion injury by inhibiting apoptosis and promoting autophagy (182).

Atorvastatin pretreatment promotes the function of exosomes derived from MSCs, which can promote angiogenesis, support the survival of cardiomyocytes, and improve cardiac function. These cardioprotective effects are partially positively regulated by lncRNA H19 (183). Morphine-pretreated serum exosomes (MPC-Exo) were co-incubated with H9c2 cardiomyocytes, which could reduce the H/R damage of cardiomyocytes, enhance cell viability, inhibit cardiomyocyte apoptosis, and exert cardiomyocyte protection. This provides a new idea and target for the study of opioid cardiomyocyte protection mechanism (184). Propofol (PPF) is an intravenous anesthetic that not only has the effect of sedative anesthesia, but also inhibits inflammation and fights oxidative stress damage in cardiomyocytes (185). A study found that PPF-pretreated endothelial exosomes (P-Exos) had a protective effect on oxidative stress injury in H9c2 cells, and demonstrated that by downregulating necrotizing apoptosis-related proteins in cardiomyocytes, avoiding damage to membrane integrity, thereby reducing cell death (186). Nicorandil is a drug with ATP-sensitive potassium channel (K^+^ ATP) activation properties (187) and has a protective effect on the heart (188, 189). Gong et al. (190) pretreated BMSC-Exos with nicorandil (MSC^NIC^-Exos). Compared with MSC-Exos, MSC^NIC^-Exos can significantly reduce the infarct area, reduce inflammation, and promote vascular regeneration. This is based on the up-regulation of miR-125a-5p by MSC^NIC^-Exos, which inhibits the TRAF6/IRF5 pathway, thereby promoting the polarization of M2 macrophages, which improves the efficacy of AMI cardiac repair. Consistent with the previously mentioned (61, 62), exosomal miR-125a-5p has a protective effect on infarcted myocardium.

In summary, traditional Chinese medicine and Western medicine can regulate exosomes of different cell origins and treat myocardial ischemia through different mechanisms and effects (Table 7).

**Table 7:** Traditional Chinese and western medicine regulates exosomes of different cell origins in the treatment of myocardial ischemia, their mechanisms, and their functions.

Source of drugs (Exosomes)	Morphology	Diameter	Exosomal markers	Component	Mechanism	Function	Experiment type	Reference
Astragaloside IV			CD81, CD63, TSG101	BMSCs		Enhance angiogenesis, inhibit apoptosis	*In vitro* and *in vivo*	(169)
Astragaloside IV	A lipid bilayer membrane structure		CD9, CD81, TSG101, HSP70, ALIX	BMSCs	miR-411/HIF-1a axis	Promote neovascularization	*In vitro* and *in vivo*	(170)
Astragalus polysaccharide	A sac-shaped vesicle with a round or oval shape and cup-shaped structure	70–150 nm	CD9, CD63, TSG101	ADSCs	PI3 K/Akt pathway	Promote angiogenesis and reduce cardiomyocyte apoptosis	*In vitro*	(171)
Tongxinluo	Cup-shaped		Alix, CD63, TSG101	BMSCs/miR-146a-5p	IRAK1/NF-κB p65 pathway	Anti-apoptosis and anti-inflammation, reduce infarct size	*In vitro* and *in vivo*	(172)
Tongxinluo	Cup-shaped	Mean: 121.6 nm	Alix, TSG101	BMSCs		Improve cardiac function, decrease cardiac function, increase angiogenesis	*In vitro* and *in vivo*	(173)
Suxiao Jiuxin pill	Grape-like clusters of vesicles	Mean: 100 nm	CD63, TSG101	CMSCs		Enhance heart repair	*In vitro*	(174)
Suxiao Jiuxin pill		Mean: 100 nm	CD63, CD81, Tsg101	CMSCs		Enhance heart repair	*In vitro*	(175)
Epigallocatechin Gallate		Exo^Nor^:97.37 ± 3.68 nm, Exo^Hypo^:116.28 ± 5.73 nm, Exo^Hypo + EGCG:^135.93 ± 4.93 nm	CD63, CD81, Hsp70, Alix, TSG101	Cardiomyocytes /miR30a		Inhibit apoptosis and autophagy	*In vitro* and *in vivo*	(176)
Ginsenoside Rh2	Round or oval bilayer lipid vesicles	Mean: 112.8 nm	CD9, CD63, TSG101	BMSCs		Increase myocardial homing ability and improve the inflammatory microenvironment	*In vitro*	(177)
Irisin			CD9, CD81, TSG101	BMSCs		Repress cardiomyocytes pyroptosis and oxidative stress response	*In vitro*	(178)
Danhong Injection	Cup-shaped	100–150 nm		ECs	miR-125b/p53 Pathway	Inhibit apoptosis	*In vitro* and *in vivo*	(179)
Ginsenoside Re				EPCs	miR-144-3p/SLC7A11	Attenuate MI/R induced ferroptosis	*In vitro* and *in vivo*	(180)
Tanshinone IIA	Round vesicles with a lipid bilayer	50–150 nm	CD9, CD63, Alix	HUVECs/miR-223-5p		Ameliorate cardiac function, reduce monocyte infiltration, and promote angiogenesis	*In vitro* and *in vivo*	(181)
Oridonin			CD63, CD81, Alix	BMSCs		Increase cell proliferation and inhibit apoptosis	*In vitro* and *in vivo*	(182)
Atorvastatin	Cup-shaped	Around 100 nm	Alix, TSG101, CD81, CD63	HUVECs/LncRNA H19		Improve cardiac function recovery, reduce infarct size and cardiomyocyte apoptosis, and promote angiogenesis	*In vitro* and *in vivo*	(183)
Morphine	Round or elliptical vesicle structure, more uniform in size	30–100 nm	CD63, HSP60	Serum		Reduce H/R damage, enhance cell viability, and inhibit cardiomyocyte apoptosis	*In vitro*	(184)
Propofol	Bilayer membranous vesicle	30–150 nm	CD63, CD9, TSG101	Endothelial cells		Inhibits oxidative stress and reduces apoptosis	*In vitro*	(186)
Nicorandil	Cup-shaped morphology		Alix, Tsg101	BMSCs/miR-125a-5p	Inhibit TRAF6/IRF5 pathway	Reduce the infarct area, inflammation, and promote vascular regeneration	*In vitro* and *in vivo*	(190)

## 9 The dilemma and future development of exosomes in diagnosis and treatment

Exosomes hold great potential for clinical diagnostic applications. Liquid biopsy is a minimally invasive, convenient, and rapid *in vitro* sampling diagnostic method. Current clinical trials use exosomes as diagnostic biomarkers based on their role in intercellular communication and disease progression, as well as the various molecules they carry, including proteins, lipids, mRNAs, and other RNA species such as lncRNAs, circ-RNAs, and miRNAs. The upregulation of exosomes miR-133a, miR-208a, miR-1, miR-499-5p, and miR-30a has been described as an early diagnosis of AMI (191). In addition to miRNAs, exosomal proteins can also serve as potential biomarkers of myocardial ischemia, such as inflammatory cascade proteins serpin C1, serpin G1, CD14, and cystatin C, are increased in exosomes in stress- induced ischemia (192). In order to identify exosomal biomarkers with high diagnostics, a large number of clinical trials need to be conducted. Although the current research is still in its infancy, with further development in the field of molecular biology, exosomes may become biomarkers for MI in the near future.

A large number of experiments have confirmed that exosomes have a potential therapeutic effect on myocardial ischemia. If exosomes are widely used as a therapeutic means in clinical practice, the production, purification, storage and targeting of exosomes are limited. At present, the main methods for extracting and purifying exosomes are Ultracentrifugation Techniques, Polymer Precipitation, Size-Based Isolation Techniques, Immunoaffinity Chromatography (IAC) (193), and Microfluidics-based isolation techniques (194). Although these extraction and purification technologies have been created, it is difficult for a single technology to solve all the problems related to high quality, high purity, high yield, and no contaminants. Watson et al. (195) reported the extraction of Extracellular vesicle (EV) using Fibercell hollow-fiber bioreactor culture, which increased the yield of EV by about 10× and greatly improved the purity. In another study, de Almeida Fuzeta et al. (196) established a serum-/xeno-free microcarrier-based culture system in a Vertical-Wheel™ bioreactor (VWBR), with a 5.7-fold increase in EV concentration and a 3-fold increase in EV number. Bioreactors have been widely used in large-scale production research. If the microcarrier or hollow fiber bioreactor is adjusted to the appropriate production conditions (temperature, area, cell volume, etc.), the yield of EV can be greatly improved. However, the specific large-scale production technology of exosomes is still under development.

Exosomes, as a promising cell-free therapy, cannot be stored for a limited period of time. The current conventional storage conditions for EV are freezing in phosphate-buffered saline (PBS) at −80 °C. Storage under this condition may lead to damage, fragmentation, and alteration of EV activity, which may alter EV biological function. Therefore, it is necessary to optimize the storage conditions of exosomes to protect their biological activity and physical properties for easy storage and clinical application. Görgens et al. (197) found that the addition of human albumin and trehalose to PBS improved the collection rate of EVs stored at minus eighty degrees for short and long periods of time and improved the biological activity after the thaw-freeze cycle. Walker et al. (198) stored EVs in a 5% sucrose solution (with 50 mM Tris, and 2 mM MgCl2), which outperformed PBS in preserving EV size distribution profiles, concentrations, biomolecular surface protrusions, and membrane proteins. The optimization of exosome storage conditions still requires in-depth research. This may not be a storage solution that can completely solve the problem of exosome storage. It may need to be combined with a variety of antifreeze solutions or other means.

As a natural drug delivery tool, exosomes are favored in clinical practice. To use exosomes as drug carriers, an effective loading strategy must be found first. There are two types of exosome loading: exogenous loading and endogenous loading (199). The exogenous loading methods of exosomes include: electroporation, incubation, ultrasound, extrusion and freeze-thaw, etc (200). These loading techniques can lead to the aggregation of its cargo and alter its physical and chemical properties, resulting in reduced loading efficiency. Endogenous loading of EV allows not only direct transfection or co-incubation of specific cargo with cells, but subsequent loading of EV by endogenous cellular mechanisms before secretion into the extracellular space; engineering of cells to stably express effective therapeutic drugs, which are then combined with methods to increase the active load of cargo molecules into EV by fusing or interacting with naturally enriched molecules in EV (201).

In order to improve the targeting of exosomes, Genetic engineering, Chemical modification, Membrane fusion and other methods are used to enhance the targeting of exosomes to therapeutic sites (202). Exosome proteins include lysosome-associated membrane glycoproteins (LAMP-1 and 2B) and tetraspan proteins such as CD63, CD9, CD81, etc., which are fused with corresponding ligands to enhance exosome-specific targeted delivery (203). Wang et al. (26) used genetic technology to fuse the ischemic myocardium-targeting peptide IMTP (CSTSMLKAC) with the exosome-enriched membrane protein (Lamp2b) for the first time to form IMTP-exosomes. They used near-infrared fluorescent tracers in *in vivo* experiments to track the targeting ability of blank exosomes and IMTP-exosomes to MI tissues. Subsequently, it was found that the fluorescence signal of IMTP-exosomes injected in the MI region was stronger, indicating that compared with blank exosomes, IMTP-engineered exosomes can preferentially target ischemic myocardium. M. Davidson et al. (204) fused a vector encoding cardiac-targeting peptide (CTP) -Lamp2b into HEK 293 cells to generate exosomes stably expressing cardiac-targeting peptide (CTP)-Lamp2b (CTP-Exo) on exosome membranes by linking glycosylation sequences, and the control group was set up to express Lamp2b only on exosome membranes (CTL-Exo). Compared with CTL-Exo, CTP-Exo increased *in vivo* delivery to mouse hearts by 15% and *in vitro* delivery by 16%. For other organ sites: liver and spleen, there was no difference in delivery between the two exosomes. This suggests that exosome targeting of cardiac tissue is enhanced by genetically modified CTP-Lamp2b. To reduce immunogenicity and regulate their own exosomes, there are research teams that have applied it to the treatment of Alzheimer's disease. Alvarez-Erviti et al. (205) used self-derived dendritic cell exosomes. First, they designed Lamp2b on dendritic cells to achieve targeting. The protein fuses with nerve cell-specific rabies virus glycoprotein (RVG) peptides, followed by electroporation to purify exosomes loaded with exogenous siRNA, and finally intravenously inject RVG-targeted exosomes to deliver GAPDH siRNA specifically to the treatment site. The results showed that the specific gene for Alzheimer's disease was knocked down. At present, there are various methods to enhance the targeting of exosomes. The targeted treatment of exosomes to ischemic myocardium can also use the endogenous loading mechanism to couple the targeted part to the exosome surface, which is expected to improve the delivery of engineered exosomes to the ischemic site. This may help to improve the therapeutic effect of targeted ischemic myocardium while optimizing the possible side effects of off-target.

Although these technologies for improving exosome extraction, purification, loading, targeting, etc. are currently immature, with the rapid development of medicine, exosomes may be used as diagnostic markers of myocardial ischemia in the near future, and exosomes can be widely used in the treatment of myocardial ischemia. Standardized procedures for exosome biomarker analysis and evaluation will also promote the development of exosome-based clinical analysis. This will be an important step towards effective and mature cardiovascular disease treatment.

## 10 Conclusions and prospects

**Figure 2:**
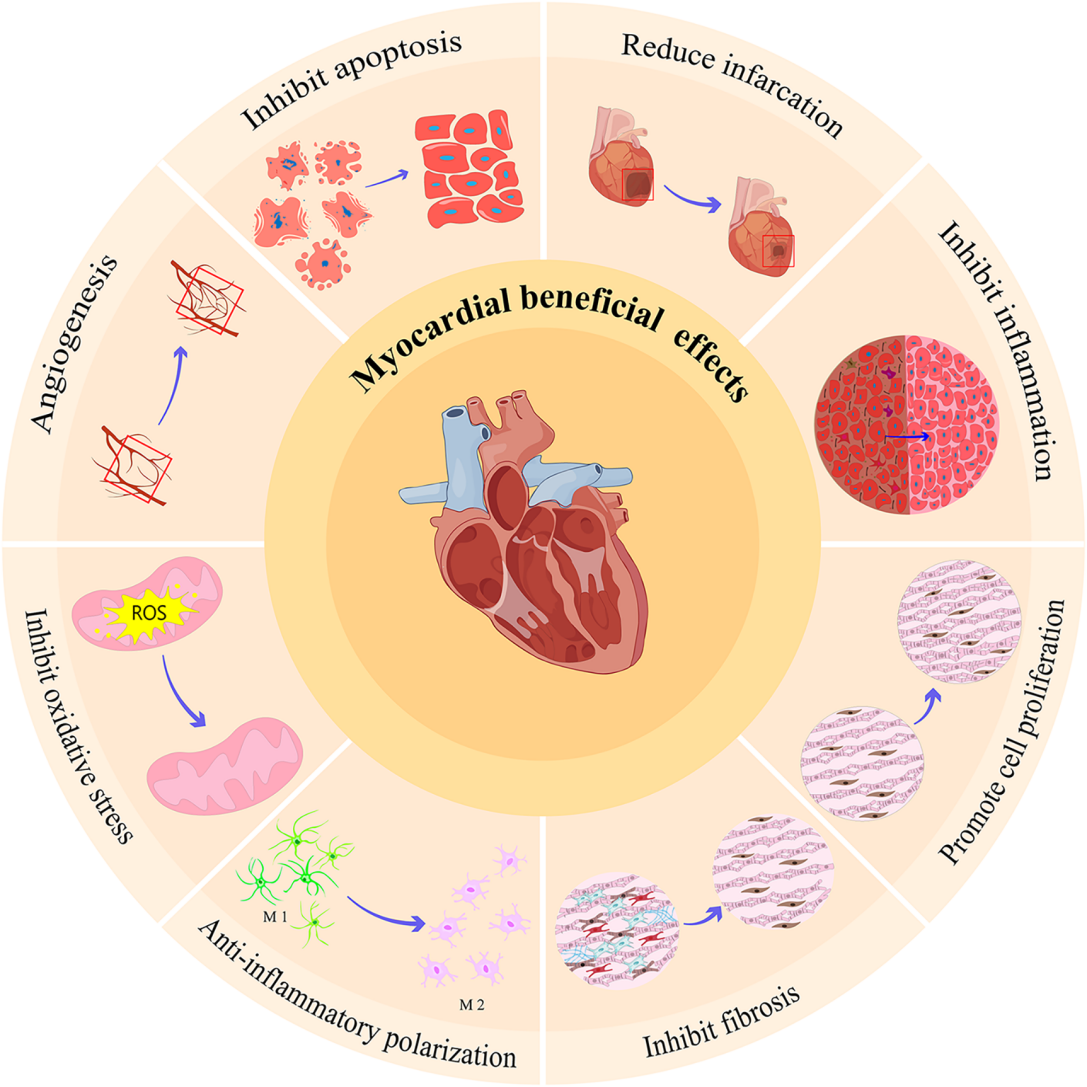
Beneficial effects of exosomes from different sources on myocardial ischemia injury. The beneficial effects of these exosomes from different sources on myocardial ischemia injury mainly include: inhibit apoptosis, promote angiogenesis, inhibit oxidative stress, promote macrophage polarization from M1 to M2, inhibit fibrosis, promote cell proliferation, inhibit inflammation, and reduce infarction.

**Figure 3:**
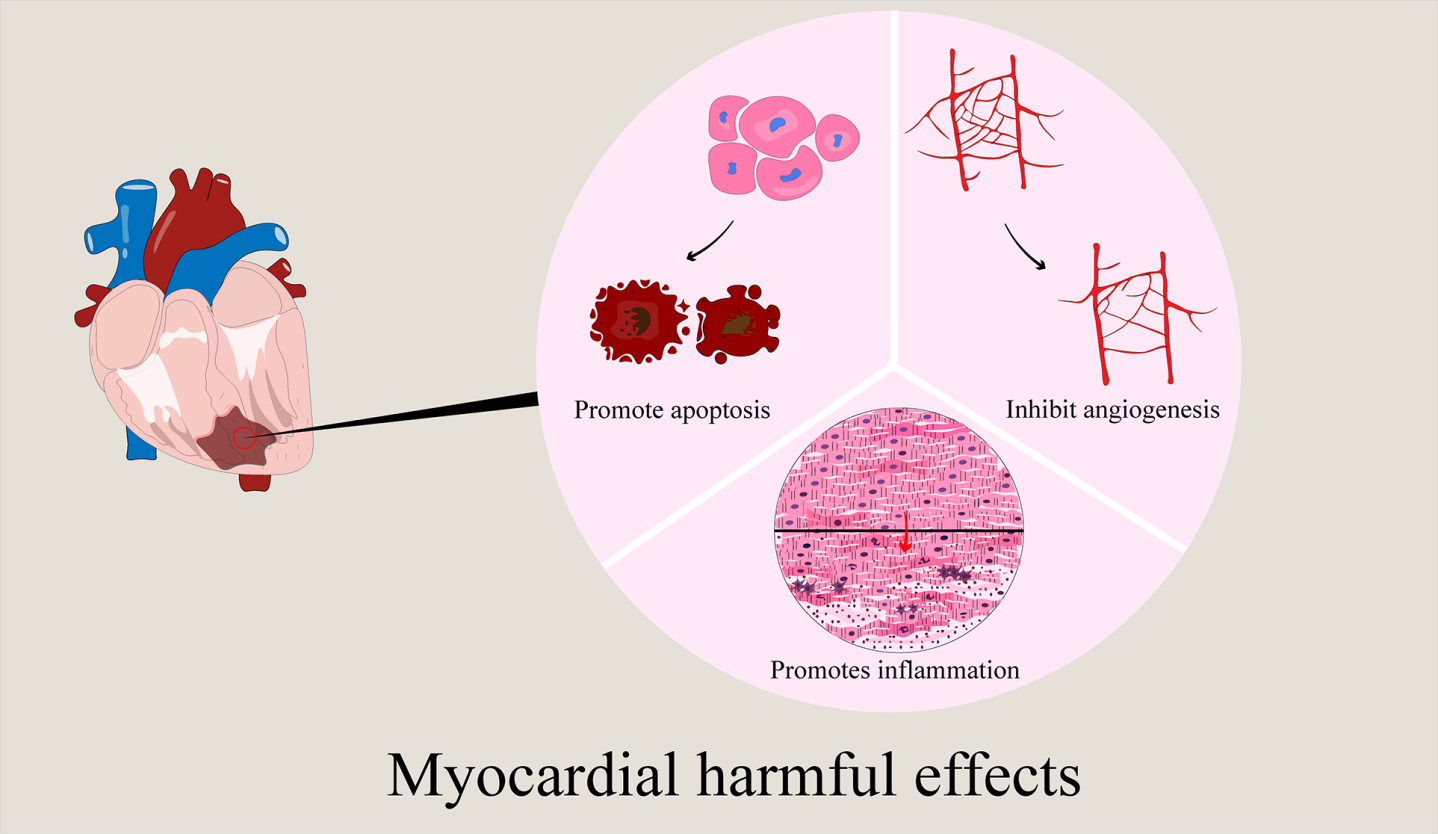
Harmful effects of exosomes from different sources on myocardial ischemia injury. The harmful effects of these exosomes from different sources on myocardial ischemia injury mainly include: promote apoptosis, inhibit angiogenesis, and promote inflammation.

**Figure 4:**
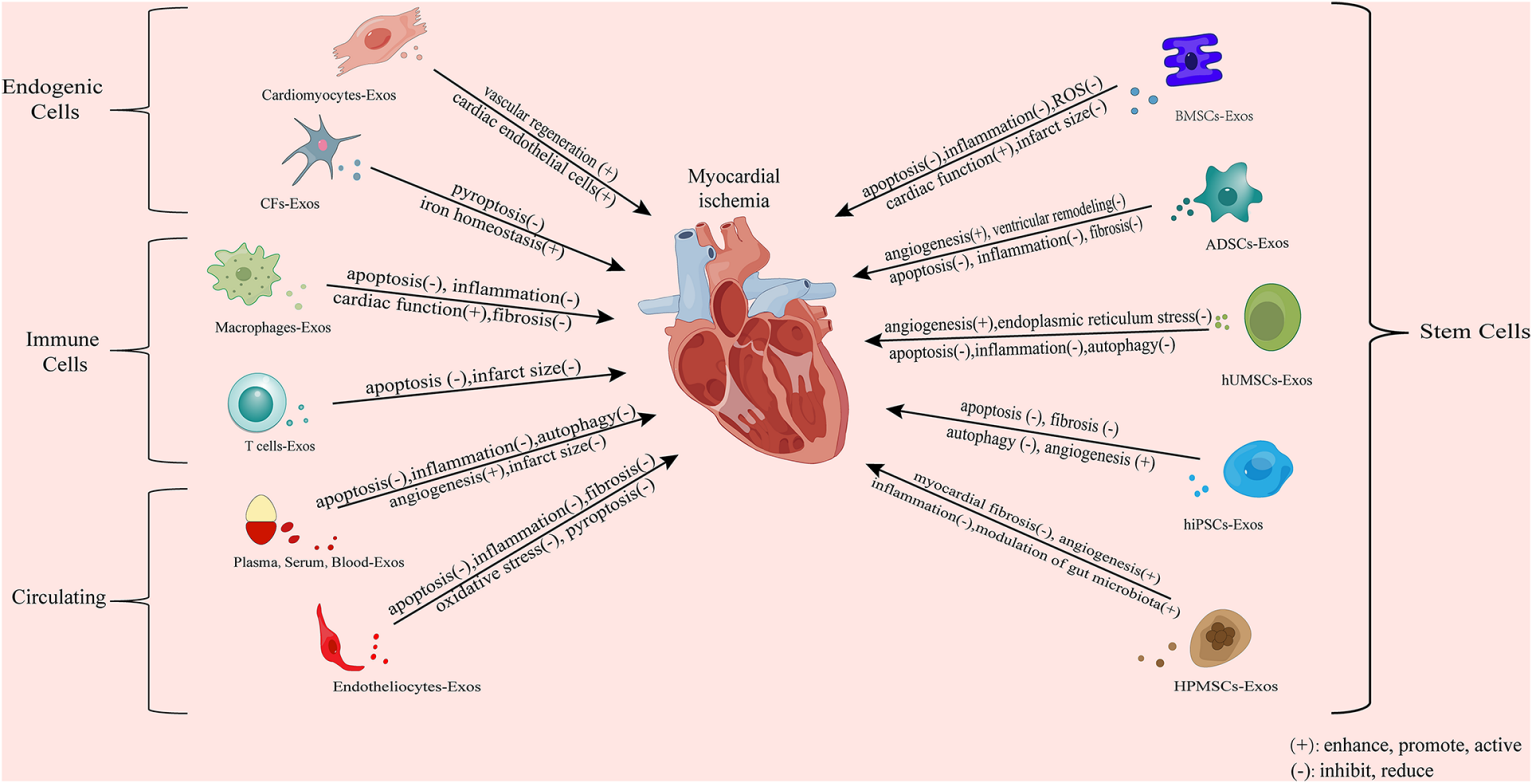
Mechanism whereby exosomes from different sources exert an effect on ischemia myocardial tissues. CFs, cardiac fibroblast; BMSCs, bone marrow mesenchymal stem cells; ADSCs, adipose-derived mesenchymal stem cells; HUMSCs, human umbilical cord mesenchymal stem cells; hiPSCs, human-induced pluripotent-stem-cell-derived exosomes. The exosomes involved in myocardial ischemia are mainly derived from the cells of the cardiovascular system and peripheral system. Exosomes from the cardiovascular system can reduce myocardial injury by promoting cardiomyocyte proliferation and angio-genesis and inhibiting pyroptosis. Exosomes from mesenchymal stem cells, including BMSCs, ADSCs, and HUMSCs, have the functions of reducing inflammation, inhibiting autophagy and pyroptosis, and promoting angiogenesis. Serum and plasma-derived exosomes and some other exosomes also actively participate in the physiological regulation process after myocardial ischemia, including inhibiting apoptosis and autophagy, exerting anti-inflammatory effects, promoting angiogenesis, and reducing myocardial infarction. In addition, exosomes derived from immune cells can improve the cardiac function of the ischemic myocardium, relieve calcium overload, and inhibit myocardial fibrosis. Induced exosomes of cellular origin can inhibit apoptosis and myocardial fibrosis, and promote angiogenesis.

To sum up, the exosomes involved in myocardial ischemia are mainly derived from the cells of the cardiovascular system and peripheral system. These exosomes from different sources play a beneficial (mainly) (Figure 2) and harmful effects (Figure 3) against myocardial ischemic injury. Exosomes from the cardiovascular system (including CFs-Exos) can reduce myocardial injury by promoting cardiomyocyte proliferation and angiogenesis, inhibiting cell pyroptosis. Exosomes from mesenchymal stem cells, including BMSCs, ADSCs, and HUMSCs, have the functions of reducing inflammation, inhibiting autophagy and pyroptosis, and promoting angiogenesis. Serum, plasma, blood-derived exosomes and some other exosomes (such as endotheliocytes) also actively participate in the physiological regulation process after myocardial ischemia, including inhibiting apoptosis, pyroptosis and autophagy, exerting anti-inflammatory effects, promoting angiogenesis, and reducing myocardial infarction. In addition, exosomes derived from immune cells (such as macrophages, dendritic cells and T-cell) can improve the cardiac function of the ischemic myocardium, relieve calcium overload, and inhibit myocardial fibrosis. Induced exosomes of cellular origin (such as hiPSC-ECs) can inhibit apoptosis and myocardial fibrosis, and promote angiogenesis (Figure 4). Furthermore, an increasing number of *in vivo* and *in vitro* experimental results confirm that when myocardial tissue is ischemic, exosomes of different cell origins can target the ischemic site and have beneficial (mostly) or harmful effects (occasionally) on ischemic myocardial tissue. Most exosomes from different sources can protect the damaged myocardium, but some experimental results show that some exosomes from cardiomyocytes can inhibit angiogenesis, promote inflammation and cardiomyocyte apoptosis (32, 37, 150, 151, 158); circulating exosomes also induce inflammation and ferroptosis, exacerbate MI/RI (142); M1 macrophages and CD4^+^ T-cell-derived exosomes can promote inflammation and ventricular remodeling (163). In addition, one study has shown that cardiomyocyte-derived exosomes also have a dual effect, both stimulating M1 macrophage polarization to induce inflammatory responses, and simultaneously targeting TRAF6 to exert anti-inflammatory effects (36). Finally, exosomes produced after drug treatment have a protective effect on ischemic myocardium, too (169–184, 186). For example, enhance angiogenesis, inhibit apoptosis and improve cardiac function, etc.

The comprehensive study of exosomes is expected to make breakthroughs in the diagnosis and treatment of myocardial ischemia, and is expected to improve the health status and quality of life of patients in the future.
